# High-Entropy Diborides—Silicon Carbide Composites by Reactive and Non-Reactive Spark Plasma Sintering: A Comparative Study

**DOI:** 10.3390/ma17030718

**Published:** 2024-02-02

**Authors:** Ekaterina Pakhomova, Giacomo Cao, Roberto Orrù, Sebastiano Garroni, Paolo Ferro, Roberta Licheri

**Affiliations:** 1 Unità di Ricerca del Consorzio Interuniversitario Nazionale per la Scienza e Tecnologia dei Materiali (INSTM), Dipartimento di Ingegneria Meccanica, Chimica, e dei Materiali, Università degli Studi di Cagliari, via Marengo 2, 09123 Cagliari, Italy; ekaterina.pakhomova@unica.it (E.P.); giacomo.cao@unica.it (G.C.); roberta.licheri@unica.it (R.L.); 2 Dipartimento di Scienze Chimiche, Fisiche, Matematiche e Naturali, Università degli Studi di Sassari, 07100 Sassari, Italy; sgarroni@uniss.it; 3 Dipartimento di Tecnica e Gestione dei Sistemi Industriali, Università di Padova, Stradella S. Nicola 3, 36100 Vicenza, Italy; paolo.ferro@unipd.it

**Keywords:** high-entropy borides, silicon carbide, spark plasma sintering, self-propagating high-temperature synthesis, resistance to oxidation, fracture toughness

## Abstract

The reactive spark plasma sintering (R-SPS) method was compared in this work with the two-step SHS–SPS route, based on the combination of the self-propagating high-temperature synthesis (SHS) with the SPS process, for the fabrication of dense (Hf_0.2_Mo_0.2_Ti_0.2_Ta_0.2_Nb_0.2_)B_2_–SiC and (Hf_0.2_Mo_0.2_Ti_0.2_Ta_0.2_Zr_0.2_)B_2_–SiC ceramics. A multiphase and inhomogeneous product, containing various borides, was obtained at 2000 °C/20 min by R-SPS from transition metals, B_4_C, and Si. In contrast, if the same precursors were first reacted by SHS and then processed by SPS under the optimized condition of 1800 °C/20 min, the desired ceramics were successfully attained. The resulting sintered samples possessed relative densities above 97% and displayed uniform microstructures with residual oxide content <2.4 wt.%. The presence of SiC made the sintering temperature milder, i.e., 150 °C below that needed by the corresponding additive-free system. The fracture toughness was also markedly improved, particularly when considering the Nb-containing system processed at 1800 °C/20 min, whereas the fracture toughness progressively decreased (from 7.35 to 5.36 MPa m^1/2^) as the SPS conditions became more severe. SiC addition was found to inhibit the volatilization of metal oxides like MoO_3_ formed during oxidation experiments, thus avoiding mass loss in the ceramics. The benefits above also likely took advantage of the fact that the two composite constituents were synthesized in parallel, according to the SHS–SPS approach, rather than being produced separately and combined subsequently, so that strong interfaces between them were formed.

## 1. Introduction

The need for structural components able to operate under harsh conditions (high temperatures, elevated heat fluxes, oxidation, corrosive environments, neutron irradiations, etc.) encountered in various application fields (aerospace, metallurgy, nuclear, etc.) prompts the development of ultra-high temperature ceramics (UHTCs) able to face such requirements [1]. To this aim, a significant effort was made to identify suitable processing techniques for their obtainment in bulk form and the related characterizations. Attention has been mostly focused on individual transition metal borides (ZrB_2_, HfB_2_, TaB_2_, etc.) and carbides (ZrC, HfC, TaC, MoC, etc.) [2,3,4]. The introduction of proper amounts of Si-containing additives (SiC, MoSi_2_, HfSi_2_, etc.) to the latter systems was found to facilitate the consolidation of their difficult-to-sinter powders, as well as concurrently improving the oxidation resistance and mechanical properties [5,6,7].

In the last decade, great attention was given to high-entropy borides (HEBs), a recently discovered sub-class of UHTCs resulting from the combination, in a near-equimolar ratio, of four to five individual metal borides, to generate single-phase crystalline solid solutions with maximum configurational entropy and, consequently, superior thermodynamic stability at high temperatures [8]. Members of HEBs were reported to show better properties as compared with their single boride constituents [8,9,10]. The experimental and computational results obtained so far have been critically examined in two recent review papers, in view of the possible exploitation of such systems for applications under extreme environments [11,12]. The need for additional studies from both the experimental and the theoretical viewpoints has been emphasized to enhance the understanding of such novel and complex UHTCs.

As for the case of individual borides, additive-free HEBs unavoidably suffer from insufficient oxidation resistance at high temperatures, as well as low fracture toughness (<4 MPa m^1/2^) [13,14,15]. Therefore, the presence of the secondary Si-containing phases mentioned above is expected to improve the latter properties also in multi-metallic diboride systems, in addition to making their powder consolidation easier. Accordingly, few studies have recently addressed the combination of some HEBs with SiC [16,17,18,19,20]. For instance, the mixture obtained after adding α-SiC to HEB powders synthesized by borocarbothermal reduction (BCR, 1600 °C/1 h/10 °Cmin^−1^/vacuum) of metal oxides with B_4_C was processed by SPS to produce (Hf_0.2_Zr_0.2_Nb_0.2_Ta_0.2_Ti_0.2_)B_2_–20 vol.%SiC [16,17]. It was found that the presence of the additive promoted sample densification, led to a refined product microstructure, and improved fracture toughness through crack deflection and branching mechanisms. Following a similar approach, dense (Hf_0.2_Zr_0.2_Mo_0.2_Nb_0.2_Ti_0.2_)B_2_–20 vol.%SiC and (Hf_0.2_Mo_0.2_Ta_0.2_Nb_0.2_Ti_0.2_)B_2_–20 vol.%SiC were prepared at 2000 °C by SPS starting from powders synthesized by either BCR or borothermal reduction (BR) [18]. It was reported that bulk ceramics from BCR powders displayed fine-grained microstructure and superior Vickers hardness, whereas higher fracture toughness corresponded to samples obtained from BR powders. The BCR route was also used to synthesize (V,Ti,Ta,Nb)B_2_–20.8 wt.%SiC powders, and the electromagnetic absorption properties of the obtained product were investigated [19]. Finally, (Hf_0.2_Zr_0.2_Nb_0.2_Ta_0.2_Ti_0.2_)–x SiC (x = 0, 10, 20, 30%vol.) was also fabricated by the BCR–SPS approach [20]. In the latter study, the introduction of SiC particles was confirmed to improve powder consolidation and inhibit HEB grain growth during SPS (1800 °C/10 min/30 MPa). Moreover, an increase in the SiC content was found to enhance the Vickers hardness (from ~19.5 to ~21 GPa) and K_IC_ values because of the synergic effect produced by grain refinement and the presence of the carbide phase.

So far, no attention has been given to the oxidation properties of HEB–SiC systems. In addition, only the few ceramic compositions mentioned above have been investigated.

In this work, the synthesis and simultaneous sintering of (Hf_0.2_Mo_0.2_Ti_0.2_Ta_0.2_Nb_0.2_)B_2_–SiC was first attempted by reactive SPS (R-SPS) starting from elemental transition metals, Si, and B_4_C. The principle of the latter approach was to chemically transform the initial reactants into the desired material during the SPS process, with the sample being concurrently densified by the direct application of a mechanical load.

Alternatively, the two-step SHS–SPS route, successfully employed for the obtainment of various quinary HEBs [10,21,22], was employed. In the latter route, the same precursors used during R-SPS were reacted by self-propagating high-temperature synthesis (SHS), and the resulting powders were subsequently processed by SPS at different temperatures and processing times. A schematic representation of the R-SPS and SHS–SPS processing approaches used in this work can be found in [23]. In particular, the SHS–SPS method was adopted for the obtainment of (Hf_0.2_Mo_0.2_Ti_0.2_Ta_0.2_Nb_0.2_)B_2_–SiC and (Hf_0.2_Mo_0.2_Ti_0.2_Ta_0.2_Zr_0.2_)B_2_–SiC systems. The densification behavior of SHS powders was analyzed and compared with those displayed by the corresponding additive-free counterparts. The mechanical properties and oxidation behavior at high temperatures of the sintered ceramics were finally evaluated and compared.

## 2. Materials and Methods

In Table 1 the characteristics of the starting powders used in this work are reported. (Hf_0.2_Mo_0.2_Ti_0.2_Ta_0.2_Me_0.2_)B_2_–SiC (with Me = Nb or Zr) was prepared by R-SPS and SHS, according to the following reaction stoichiometry:

0.4 Hf + 0.4 Mo + 0.4 Ti + 0.4 Ta + 0.4 Me + Si + B_4_C → 2(Hf_0.2_Mo_0.2_Ti_0.2_Ta_0.2_Me_0.2_)B_2_ + SiC (1)

which correspond approximately to (Hf_0.2_Mo_0.2_Ti_0.2_Ta_0.2_Nb_0.2_)B_2_–27.7 vol.%SiC and (Hf_0.2_Mo_0.2_Ti_0.2_Ta_0.2_Zr_0.2_)B_2_–27.4 vol.%SiC, respectively. For the sake of brevity, the two composite systems will be hereafter indicated as HEB_Nb–SiC and HEB_Zr–SiC.

About 20 g of powder was mixed for 20 min in a plastic vial equipped with 10 zirconia balls using a Horizontal Roller Ball Mill (mod. BML-2, Witeg Labortechnik GmbH, Wertheim am Mein, Germany). The resulting mixtures were processed by either reactive SPS or SHS. In the latter case, about 7 g of the mixture was cold pressed to form cylindrical pellets (10 mm diameter, 20–22 mm height) to be reacted by SHS inside a stainless steel chamber first evacuated and then filled with argon. An electrically heated tungsten filament was used to activate the synthesis reaction. Further details of SHS experiments can be found elsewhere [24]. The reacted products received a 20 min ball milling treatment in a SPEX 8000 (SPEX CertiPrep, Metuchen, NJ, USA) mixer/mill, with a stainless steel vial and steel balls (the ball to powder weight ratio was equal to 2). A laser light scattering analysis (CILAS 1180, Orléans, France) was employed to determine the particle size of the resulting powders. This analysis was based on Fraunhofer’s theory and was conducted in wet mode, utilizing water as the dispersing agent. Prior to each analysis, a 60 s sonication process was applied to disperse the particles effectively. For each composition under investigation, three distinct samples were collected for laser diffraction analysis. Within each sample, three measurements were carried out, with the stirrer/pump and sonication maintained consistently between subsequent measurements. The time interval between two consecutive measurements was set at 60 s.

The sintering process was performed using an SPS apparatus (515S model, Fuji Electronic Industrial Co., Ltd., Sagamihara, Japan) under vacuum conditions (about 20 Pa). Approximately 3.5 (HEB_Zr–SiC) or 3.7 g (HEB_Nb–SiC) of powders was placed inside a cylindrical graphite die (30 mm external diameter; 15 mm inside diameter; 30 mm height) equipped with two punches (14.7 mm diameter, 20 mm height). SPS runs were conducted under a temperature-controlled mode using an infrared pyrometer (CHINO, mod. IR-AHS2, Tokyo, Japan) focused on the lateral surface of the die. The temperature was increased at a constant rate (HR = 200 °C/min) from the room value to the maximum level (T_D_). The sample was then maintained at T_D_ for a prescribed duration, in the range 5–20 min. The effect of T_D_ on the density and composition of the SPS product was investigated in the range 1500–1900 °C. A mechanical pressure of 20 MPa was applied during the entire experiment duration. For the sake of reproducibility, each experiment was repeated at least twice.

The same sintering equipment, ancillary devices, and die/plungers mentioned above were also used for directly processing the initial precursors listed in Table 1 by reactive SPS according to reaction (1). A relatively higher holding temperature (T_D_ = 2000 °C) was adopted in the latter case, compared with that considered with the SHS–SPS approach, while the heating rate was lowered (HR = 100 °C/min instead of 200 °C/min). The dwell time at T_D_ and the applied pressure were set to 20 min and 20 MPa, respectively.

Before characterization, bulk products obtained by SHS–SPS or R-SPS were cut, ground, and polished using progressively finer abrasive paper. The Archimedes method was employed to evaluate their absolute densities using distilled water as the immersing medium. The corresponding relative densities were calculated by considering the theoretical values of 7.15 g/cm^3^ (HEB_Nb–SiC) and 7.06 g/cm^3^ (HEB_Zb–SiC) for the composite systems. The latter ones were evaluated by applying a rule of mixture [25] and using 8.67 g/cm^3^ (HEB_Nb) [22], 8.52 g/cm^3^ (HEB_Zr) [22], and 3.21 g/cm^3^ (SiC) [26] as theoretical densities for the individual ceramic constituents.

The phase identification and structural characteristics of the SHS powder, R-SPS, and SHS–SPS samples were determined by X-ray diffraction analysis (rotating anode SmartLab Rigaku, Akishima-shi, Japan, equipped by a NaI (Tl) Sodium Iodide Scintillation Detector, and Bruker D8 Advance, Leipzig, Germany, equipped with multimode LYNXEYE XE-T detector) using Cu K_α_ radiation, over a range of scattering angles 2 ϑ from 10 to 130, in steps of 0.05° with 15 s acquisition time per angle. The phase amounts and microstructural parameters were estimated with the Rietveld method by analyzing the XRD patterns with the MAUD program [27].

High-resolution scanning electron microscopy (HRSEM) (mod. S4000, Hitachi, Tokyo, Japan) equipped with an UltraDry EDS Detector (Thermo Fisher Scientific, Waltham, MA, USA) was used to observe the microstructure, as well as to verify compositional homogeneity in the sintered samples.

Oxidation tests on sintered samples were performed in air using a muffle furnace (LT 24/11/B410, Nabertherm, Lilienthal, Germany). During these experiments, samples were heated at a rate of 5 °C/min from room temperature to a maximum value, in the range of 600–1300 °C, followed by an isothermal step of 1 h duration. The composition and the microstructure of the treated specimens were then examined by XRD and SEM.

Mechanical properties were determined by means of Micro Vickers Hardness Testers FUTURE-TECH FM-810 (Kawasaki, Kanagawa 210-0804, Japan). Samples were embedded into phenolic resin and then lapped and polished. A load of 1 N was applied with a loading time of 15 s. At least five measurements were performed for each sample, and the average values were then calculated. The fracture toughness was evaluated using a load of 1 N in order to cause cracks to propagate from the indent tips. The scanning electron microscope (SEM) Quanta 400 of the Field Electron and Ion Company (Hillsboro, OR, USA) was used to determinate the crack length.

The fracture toughness was then calculated based on crack lengths, according to Evans’ and Charles’ equation [28,29], namely,

(2) $$K_{\mathrm{IC}}=0.0824\;\frac{P}{c^{3/2}}$$

where K_IC_ is the fracture toughness, *P* is the load, and *c* is the average crack length measured from the indentation center.

## 3. Results and Discussion

### 3.1. Reactive Spark Plasma Sintering Route

**Figure 1:**
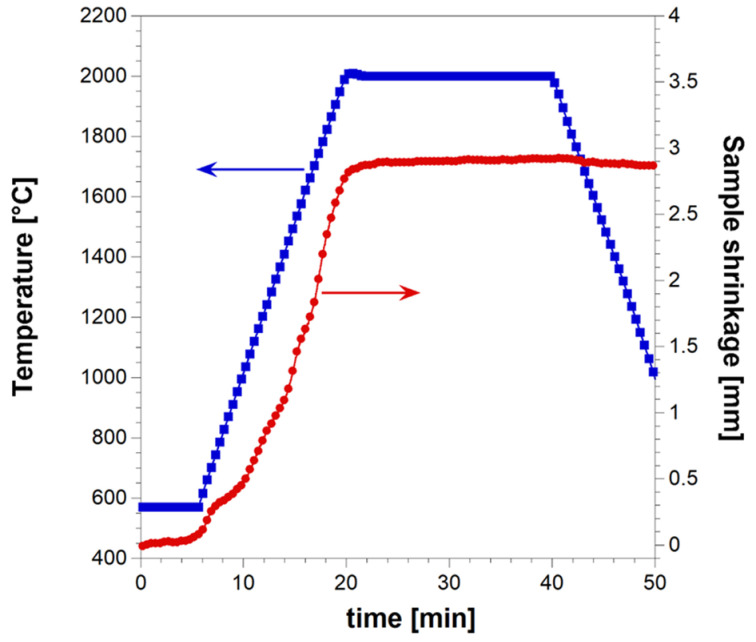
Temporal profiles of temperature (blue curve) and sample shrinkage (red curve) recorded during the preparation of dense HEB_Nb–SiC by reactive SPS (T_D_ = 2000 °C, HR = 100 °C/min, t_D_ = 20 min, P = 20 MPa).

The simultaneous synthesis and consolidation of HEB_Nb–SiC by R-SPS were first attempted. This approach was successfully adopted in the literature for the preparation of dense ZrB_2_–SiC from Zr, B_4_C, and Si [30]. When the starting reactants were processed for 20 min by SPS at T_D_ = 1900 °C, with a heating rate of about 200 °C/min, a nearly full dense ceramic composite (relative density >99.5%) was correspondingly obtained. To the same aim, the powder mixture consisting of initial precursors according to Reaction (1) with Me = Nb was processed for 20 min by SPS at 2000 °C with a non-isothermal heating time (t_H_) of 20 min (heating rate of 100 °C/min). The more severe sintering temperature (2000 °C) considered here compared with that (1900 °C) used for the preparation of ZrB_2_–SiC was to facilitate the diffusion of the different metals contained in the HEB matrix across the material volume. The relatively lower heating rate applied in the present work also contributed to achieve the same purpose. In addition, such a condition was also aimed to avoid the occurrence of the exothermic synthesis reaction (1) under the combustion mode. Indeed, as demonstrated in previous works [31,32], the latter regime, which is undesirable due to a series of negative drawbacks (residual porosity, not homogeneous products, die/plunger breakage, safety problems, etc.), is more likely established when operating at relatively higher heating rates. The sample shrinkage time profile recorded during the R-SPS experiment is plotted in Figure 1 along with the imposed temperature pattern. It is seen that powder sintering took place in a gradual manner, mostly during the non-isothermal heating step. When the T_D_ value was reached, sample shrinkage further increased albeit at a much slower rate. Based on the sintering curve shown in Figure 1, the occurrence of combustion-like reactions could be readily excluded since a sudden change in the sample shrinkage did not take place. A similar behavior was encountered during the preparation of ZrB_2_–SiC by R-SPS, with the initial reactants gradually transformed to the final composite until the complete conversion was attained [30].

The absolute density of the HEB_Nb–SiC-based ceramic produced in this work was 6.88 ± 0.12 g/cm^3^, corresponding to a relative density of 96.2 ± 1.6%, which was evaluated by considering 7.15 g/cm^3^ as the theoretical/reference value.

**Figure 2:**
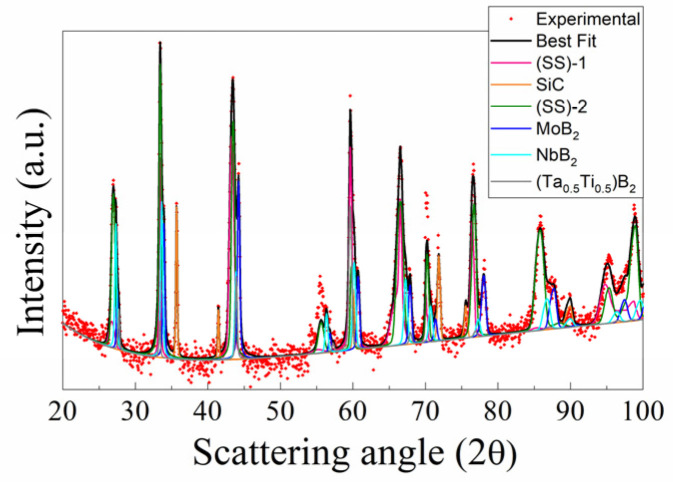
XRD pattern and related Rietveld refinement (R_wp_ = 9.6%) of HEB_Nb–SiC product obtained by reactive SPS (T_D_ = 2000 °C, HR = 100 °C/min, t_D_ = 20 min, P = 20 MPa) according to reaction (1) with Me = Nb. (SS)-1 and (SS)-2 are two solid solutions of (Hf_0.2_Mo_0.2_Ti_0.2_Ta_0.2_Nb_0.2_)B_2_, with slightly different cell parameters (cf. Supplementary Table S3).

The X-ray diffraction pattern, experimental and the best fit, of the R-SPSed product is reported in Figure 2. The corresponding structural and microstructural parameters estimated by the Rietveld analysis are summarized in Supplementary Table S1. The resulting sample is characterized by a mixture of phases which consist, according to the performed Rietveld analysis (R_wp_ = 9.6%), of two solid solutions of (Hf_0.2_Ta_0.2_Nb_0.2_Mo_0.2_Ti_0.2_)B_2_, with slightly different cell parameters, and SiC, while also displaying other binary and individual diborides, namely, (Ta_0.5_Ti_0.5_)B_2_, MoB_2_, and NbB_2_. The total amount of undesired secondary phases resulted to be above 40 wt.% (Table S1), so that the obtainment by reactive SPS of the desired HEB–SiC product was far from being achieved.

**Figure 3:**
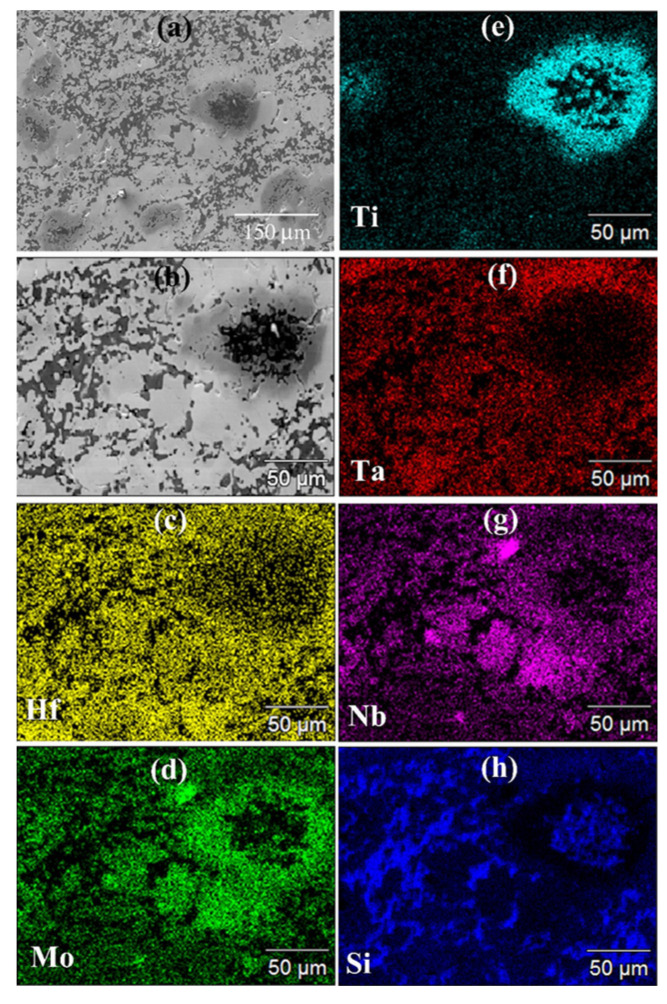
General (**a**) and detailed (**b**) SEM views along with the corresponding Hf (**c**), Mo (**d**), Ti (**e**), Ta (**f**), Nb (**g**), and Si (**h**) EDS maps of the HEB_Nb–SiC sample obtained by R-SPS (T_D_ = 2000 °C, HR = 100 °C/min, t_D_ = 20 min, P = 20 MPa) according to Reaction (1) with Me = Nb.

The SEM/EDS observations (cf. Figure 3) were consistent with the XRD outcomes. The SEM micrograph confirmed the high densification level reached during the process. However, the same micrograph and the corresponding elemental EDS maps also evidenced that the sintered sample displayed a very inhomogeneous microstructure. It was then possible to conclude that as for the additive-free HEB_Nb system investigated by Tallarita et al. [10], and despite the relatively higher temperature (2000 °C) adopted in this work, the R-SPS approach did not provide the required conditions for synthesizing the corresponding HEB–SiC ceramic.

### 3.2. SHS–SPS Route

#### 3.2.1. The (Hf_0.2_Mo_0.2_Ti_0.2_Ta_0.2_Nb_0.2_)B_2_–SiC System

As described in Section 3.1, the attempt to perform the direct synthesis and sintering of HEB_Nb–SiC by R-SPS failed. To achieve such a goal, these two steps were then conducted separately, using the SHS–SPS approach. During the first processing stage, precursors, combined according to Reaction (1) with Me = Nb, were first reacted by SHS. As for the synthesis of additive-free (Hf_0.2_Mo_0.2_Ti_0.2_Ta_0.2_Nb_0.2_)B_2_ from elemental reactants [10], also the exothermic Reaction (1) displayed a self-sustaining character. The observed behavior was like that seen with the preparation of ZrB_2_–SiC [30] and HfB_2_–SiC [33] from Zr or Hf, B_4_C, and Si. On the other hand, a preliminarily mechanical treatment was required to induce the SHS reaction in the TaB_2_–SiC system [11]. The latter studies also evidenced that when a single transition metal was involved, such a synthesis technique was able to lead to the desired MeB_2_–SiC product with no secondary phases.

The transformation of the initial reactants to the desired HEB_Nb and SiC phases was verified by examining the SHS product by XRD and SEM.

**Figure 4:**
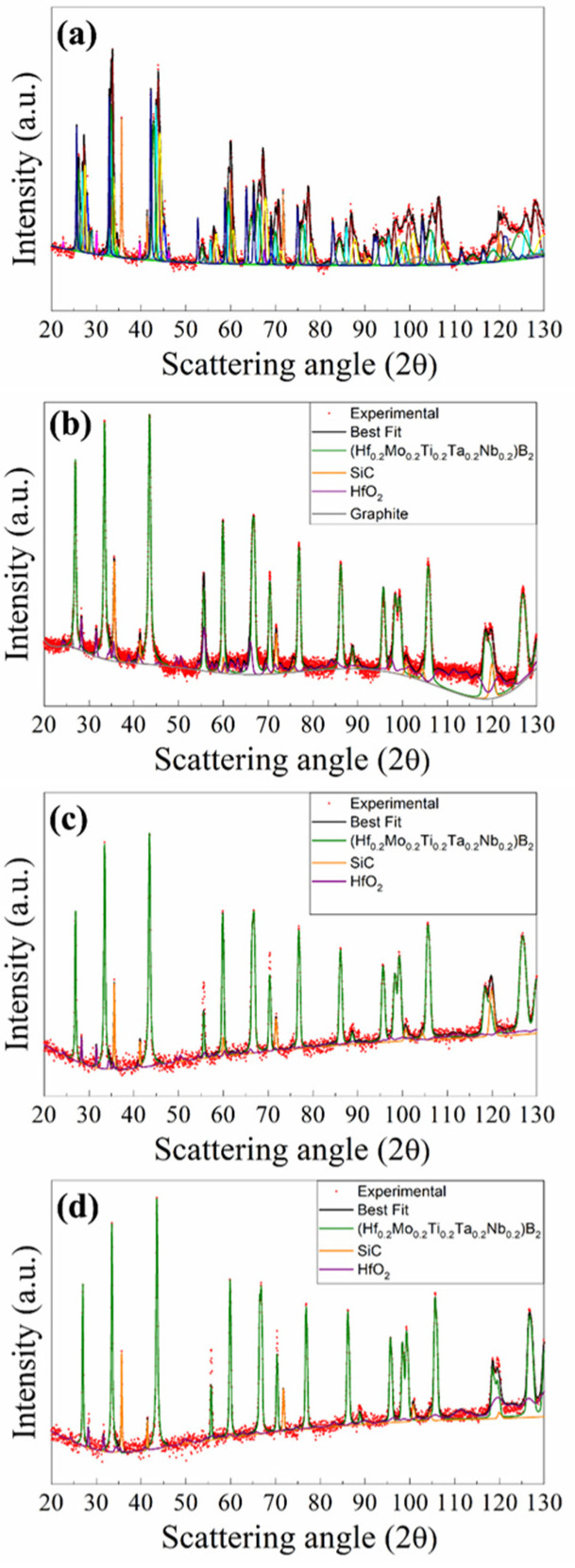
XRD patterns and related Rietveld refinement of (**a**) HEB_Nb–SiC powders obtained by SHS (R_wp_ = 9.8%) and corresponding bulk samples obtained at different SPS conditions (20 MPa, HR = 200 °C/min): (**b**) 1800 °C/20 min (R_wp_ = 7.6%), (**c**) 1900 °C/5 min (R_wp_ = 8.4%), and (**d**) 1900 °C/20 min (R_wp_ = 9.8%). (**a**) Experimental: red dots; Best Fit: dark solid line; (Hf_0.2_Mo_0.2_Ti_0.2_Ta_0.2_Nb_0.2_)B_2_: olivine solid line; TaB_2_: brown solid line; NbB_2_: pale blue solid line; SiC: orange solid line; MoSi_2_: magenta solid line; MoB_2_: blue solid line; (MoTiB_4_)_0.5_: violet solid line; TiB_2_: yellow solid line; HfB_2_: Prussian blue solid line; SiO_2_: aqua green; C (Graphite): grey solid line; Si: dark yellow solid line; B_4_C: light green solid line.

The XRD results are shown in Figure 4a, while the related structural and microstructural parameters are reported in Supplementary Table S2. As revealed by the Rietveld refinement, SHS powders presented a multiphase product, made of the desired (Hf_0.2_Ta_0.2_Nb_0.2_Mo_0.2_Ti_0.2_)B_2_ solid solution (14.9 wt.%), and SiC (11.5 wt.%) phases, with various additional metal borides, namely, HfB_2_ (9.0 wt.%), TaB_2_ (14.6 wt.%), NbB_2_ (12.8 wt.%), TiB_2_ (13.4 wt.%), MoB_2_ (9.5 wt.%), and (MoTiB_4_)_2_ (3.9%). Furthermore, lower amounts (less than 3.0 wt.%) of MoSi_2_, C, Si, SiO_2_, and B_4_C were also detected.

Table 2 reports particle size analysis results of the powders after ball milling the SHSed samples. Fine particles, with average sizes slightly larger than 2 µm, were produced. These powders were also characterized by SEM, and the corresponding results are reported in Supplementary Figure S1. The obtainment of few-micrometer-sized particles was confirmed. Moreover, the EDS map results agreed with the XRD analysis outcomes, with transition metals, particularly Ti, Nb, and Mo, not homogeneously distributed in the powders, to indicate that the synthesis of the desired phases was not achieved by SHS. As observed in previous works focused on the fabrication of various additive-free HEBs [21,22], the SHS process evolved too rapidly (a few seconds) to allow for the complete diffusion of the five metallic constituents across the sample.

**Figure 5:**
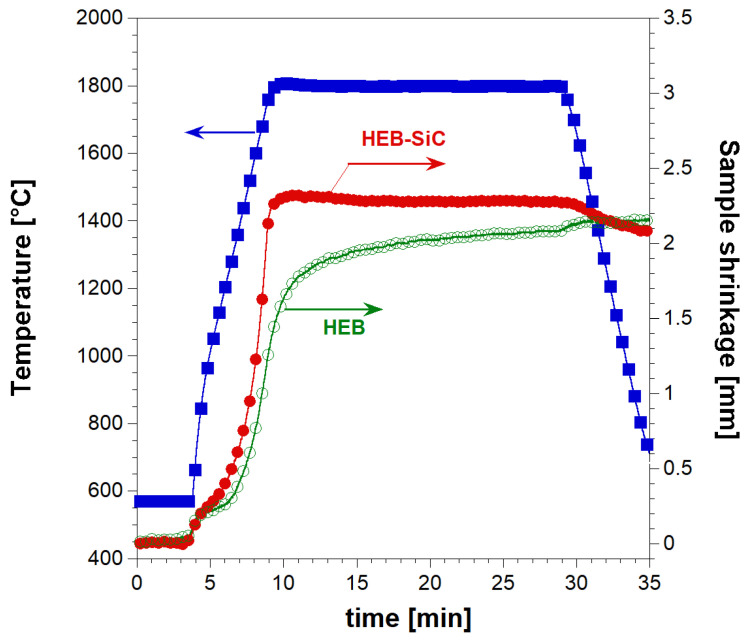
Comparison of sample shrinkage recorded during the preparation of dense HEB_Nb by SHS–SPS in the presence of (red curve) or without (green curve) SiC (T_D_ = 1800 °C, HR = 200 °C/min, t_D_ = 20 min, P = 20 MPa). The corresponding temperature profile (blue curve) is also shown.

Let us now consider the sintering step. The sample shrinkage time profile recorded during SPS is compared in Figure 5 with data obtained when the SiC-free HEB_Nb based powders, prepared according to Barbarossa et al. [22], were consolidated using the same operating parameters (T_D_ = 1800 °C, HR = 200 °C/min, t_D_ = 20 min, P = 20 MPa). The markedly higher densification rate and the superior final sample shrinkage observed with the SiC containing powders confirmed the role played by the additive as a sintering aid. It was also observed that under such conditions, the consolidation of HEB_Nb–SiC was basically confined to the non-isothermal step, whereas only negligeable changes were manifested in sample shrinkage at the dwell temperature. In contrast, additive-free powders continued their densification, albeit at a slower rate, also during the isothermal stage.

The benefit determined by the presence of SiC was also proven by the relative densities of the final products, i.e., 91.9 ± 0.09% (HEB_Nb) and 98.7 ± 0.7% (HEB_Nb–SiC), which were determined by considering 8.67 [22] and 7.15 g/cm^3^ as theoretical/reference values, respectively.

**Figure 6:**
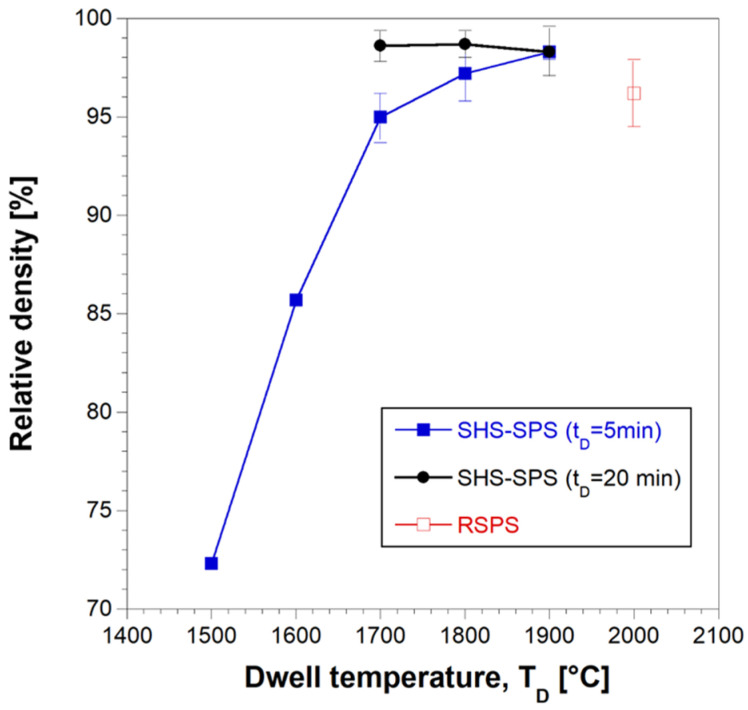
Effect of the dwell temperature on the relative density bulk HEB_Nb–SiC samples obtained by SHS–SPS when t_D_ = 5 and 20 min (HR = 200 °C/min, P = 20 MPa). Data obtained with the R-SPS approach are also reported.

The effect of the dwell temperature on product density is reported in Figure 6 for two holding time values (5 and 20 min). The extremely low densification level achieved at 1500 °C/5 min (72.3%) was progressively improved up to 98.3% as the sintering temperature was increased to 1900 °C. Similar relative densities were also achieved at lower temperatures (1700 °C), when the holding time was prolonged to 20 min. In the latter case, a further increase in T_D_ to 1800 and 1900 °C did not determine additional beneficial effects, at least from the sample densification viewpoint.

The XRD patterns of bulk samples sintered at different T_D_ values are shown in Supplementary Figure S2a,b for the cases of t_D_ equal to 5 and 20 min, respectively. The product composition was gradually improved as the holding temperature was progressively raised. The presence of secondary phases was detected in SPS products obtained when operating at 1700 °C or lower dwell temperatures. Samples prepared at 1800 °C/20 min, 1900 °C/5 min, and 1900 °C/20 min were more accurately analyzed by XRD using the Rietveld analytical procedure. The obtained results are reported in Figure 4b–d and Supplementary Table S3. The sample obtained at 1800 °C/20 min was characterized by a single solid solution phase (83.1 wt.%), together with SiC (13.6 wt.%), mainly formed during the SHS reactions. Small amounts of HfO_2_ and traces of C (2.4 and 0.9 wt.%, respectively) were also detected. Therefore, the secondary metal diborides found in the synthesized powders were all completely transformed into the expected HEB_Nb phase during SPS. Furthermore, the metal silicide phase (MoSi_2_) also produced by SHS as a consequence of the chemical interaction of Si and Mo precursors (cf. reaction (1)) was not detected in the SPS product. The obtainment of the desired HEB_Nb–SiC composite was further verified in samples processed at 1900 °C/5 min and 1900 °C/20 min. Moreover, the obtained lattice parameter values for the HEB phase, i.e., a = 3.0887–3.0891 Å and c = 3.3027–3.3035 Å (Table S3), were in line with those reported in previous works for the same system [8]. The Rietveld analysis (Table S3) also evidenced that the crystallite size of the produced HEB phase progressively increased as the sintering conditions became more severe, i.e., about 109 nm (1800/20 min), 132 nm (1900/5 min), and 152 nm (1900, 20 min).

**Figure 7:**
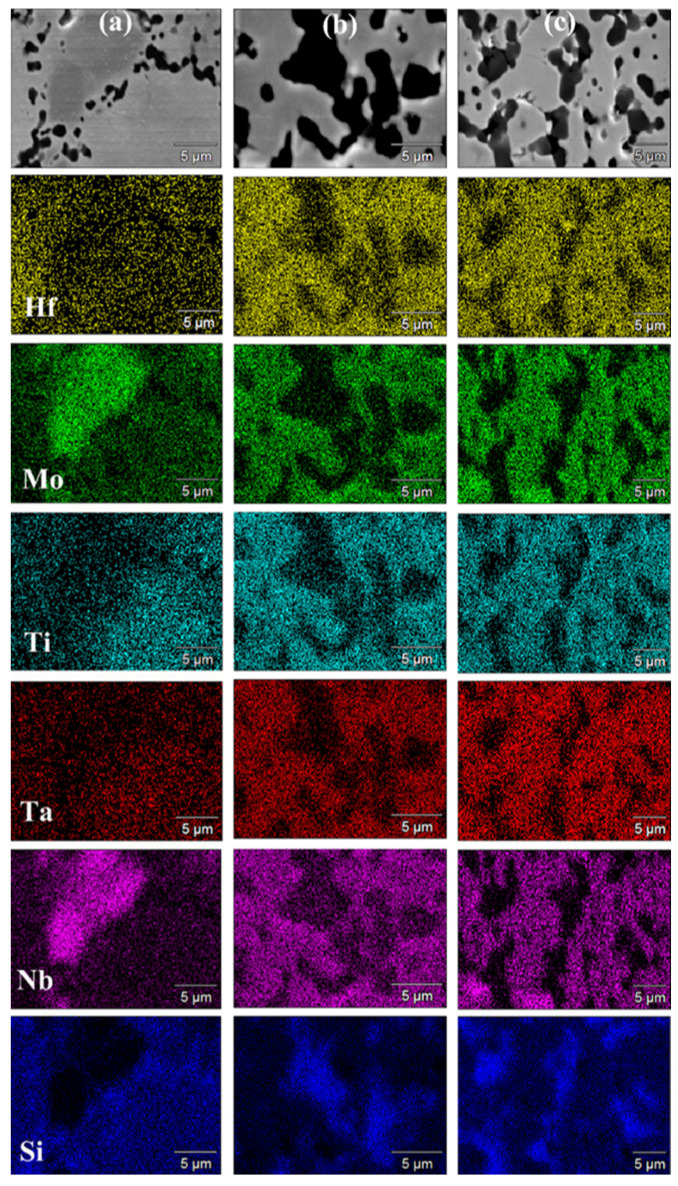
SEM micrographs and related elemental EDS maps of HEB_Nb–SiC samples obtained by SPS at t_D_ = 5 min for different T_D_ values (P = 20 MPa, HR = 200 °C/min) from SHS powders: (**a**) 1700 °C, (**b**) 1800 °C, and (**c**) 1900 °C.

**Figure 8:**
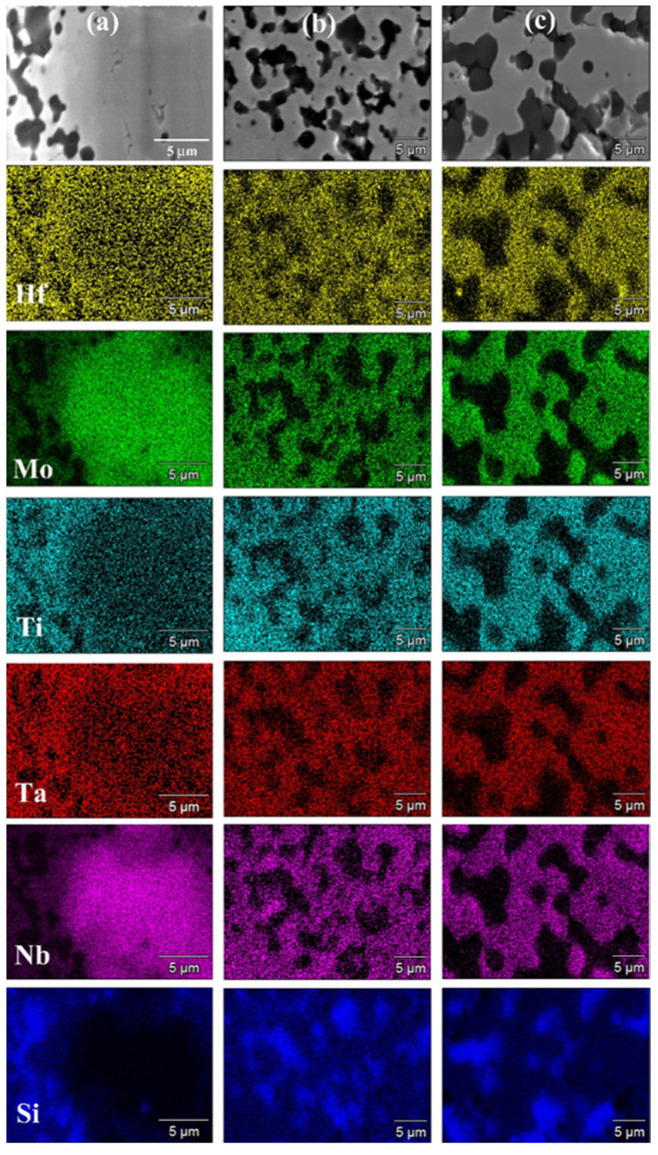
SEM micrographs and related elemental EDS maps of HEB_Nb–SiC samples obtained by SPS at t_D_ = 20 min for different T_D_ values (P = 20 MPa, HR = 200 °C/min) from SHS powders: (**a**) 1700 °C, (**b**) 1800 °C, and (**c**) 1900 °C.

SEM micrographs and corresponding elemental EDS maps shown in Figure 7 (t_D_ = 5 min) and Figure 8 (t_D_ = 20 min) are consistent with the XRD analysis results. Indeed, a T_D_ value of 1700 °C was not sufficient to adequately promote diffusion phenomena in the sample undergoing SPS. Under such conditions, an extension of the holding time from 5 min (Figure 7a) to 20 min (Figure 8a) did not produce appreciable improvements. On the other hand, as the temperature was raised to 1800 °C (Figure 7b and Figure 8b) and 1900 °C (Figure 7c and Figure 8c), significant gains in the product microstructure were correspondingly achieved.

#### 3.2.2. The (Hf_0.2_Mo_0.2_Ti_0.2_Ta_0.2_Zr_0.2_)B_2_–SiC System

**Figure 9:**
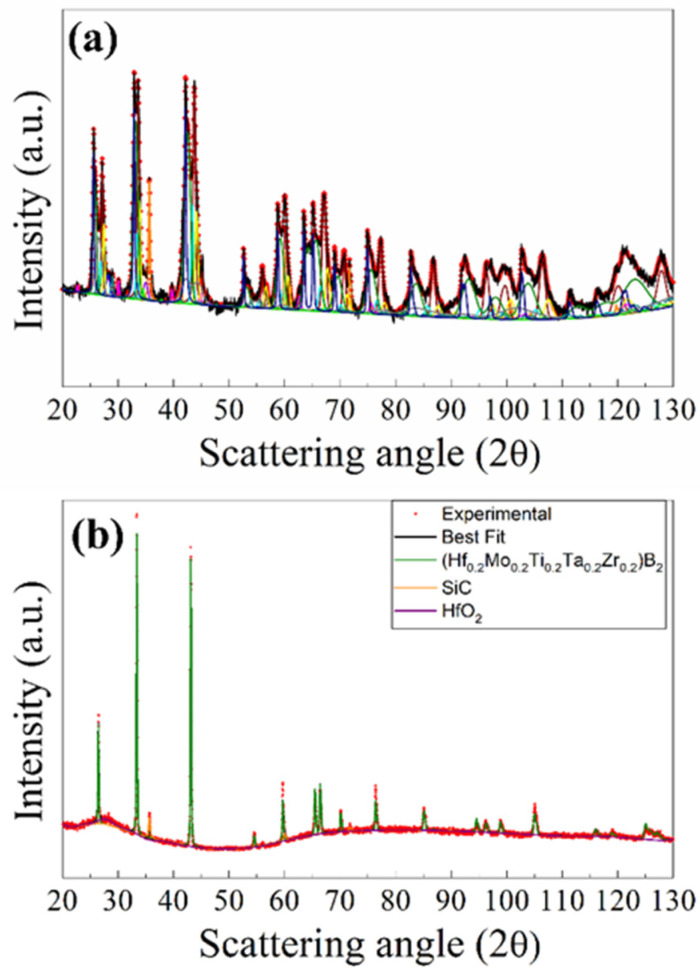
XRD patterns and related Rietveld refinement of (**a**) HEB_Zr–SiC powders obtained by SHS (R_wp_ = 10.1%) along with the corresponding SPS product (**b**) obtained at T_D_ = 1800 °C, HR = 200 °C/min, t_D_ = 20 min, P = 20 MPa (R_wp_ = 9.9%). (**a**) Experimental: red dots; Best Fit: dark solid line; (Hf_0.2_Mo_0.2_Ti_0.2_Ta_0.2_Nb_0.2_)B_2_: olivine solid line; TaB_2_: brown solid line; NbB_2_: pale blue solid line; SiC: orange solid line; MoSi_2_: magenta solid line; MoB_2_: blue solid line; (MoTiB_4_)_0.5_: violet solid line; TiB_2_: yellow solid line; HfB_2_: Prussian blue solid line; SiO_2_: aqua green; C (Graphite): grey solid line; Si: dark yellow solid line; B_4_C: light green solid line.

Based on the experimental findings attained with the HEB_Nb–SiC system, the preparation of bulk (Hf_0.2_Mo_0.2_Ti_0.2_Ta_0.2_Zr_0.2_)B_2_–SiC was performed by SHS–SPS. Initial precursors (cf. Equation (1) with Me = Zr) were reacted by SHS for the preparation of powders that were subsequently densified by SPS at 1800 °C (20 min/20 MPa), i.e., the optimal T_D_ value that allowed us to produce the desired Nb containing the ceramic composite. The resulting sintered samples possessed a relative density equal to 97.7 ± 0.6%. As for the composition, also in this case, the SHS reaction (1) did not go to completion, with the synthesized powders consisting of multiple phases. The latter ones were identified by XRD, and the corresponding content, estimated by Rietveld analysis, is reported in Figure 9a and Supplementary Table S4. Other than the prescribed HEB (11.9 wt.% only) and SiC (10.9 wt.%) phases, the SHS product was rich in various individual and binary diborides (ZrB_2_, TaB_2_, HfB_2_, MoB_2_, TiB_2_, and (MoTi)B_4_) along with other undesired species (MoSi_2_, SiO_2_, C, Si, and B_4_C).

However, as shown in Figure 9b, the complete conversion of the secondary phases into the desired ceramic composite was attained during SPS. Specifically, as evidenced by the Rietveld analysis (Supplementary Table S4), the sintered sample basically consisted of (Hf_0.2_Mo_0.2_Ti_0.2_Ta_0.2_Zr_0.2_)B_2_ (84.4 wt.%) and SiC (14.1 wt.%), with only a small amount of HfO_2_ (1.5 wt.%). As for the case of the HEB_Nb system, it was also confirmed that no silicide phases were found in the sintered samples, the initial Si precursor being finally converted into the corresponding carbide. The lattice parameter values of the HEB_Zr phase, i.e., a = 3.0976 Å and c = 3.3628 Å (Table S4), obtained by the Rietveld refinement, were in line with the literature data reported for the same system [34].

**Figure 10:**
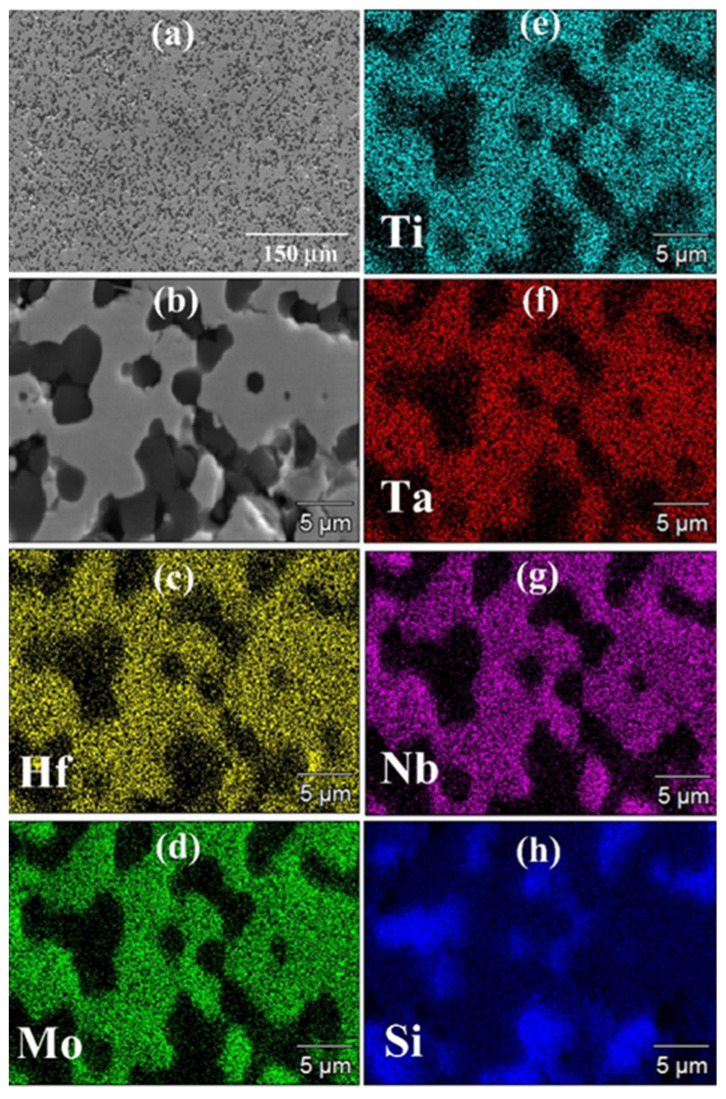
General (**a**) and detailed (**b**) SEM views along with the corresponding Hf (**c**), Mo (**d**), Ti (**e**), Ta (**f**), Nb (**g**), and Si (**h**) EDS maps of the HEB_Zr–SiC sample obtained by SPS (T_D_ = 1800 °C, t_D_ = 20 min, P = 20 MPa, HR = 200 °C/min) from powders synthesized by SHS according to Reaction (1) with Me = Zr.

The SEM/EDS observations were in accordance with the XRD analysis. In the latter regard, Figure 10 evidences that all metallic elements are very homogeneously distributed into the HEB matrix, with the secondary phase uniformly dispersed across the sample volume. SEM micrographs also testified to the high densification level achieved after SPS, with some surface porosity mostly due to grain pulling out occurring during the polishing procedure. Also in this case, it should be noted that the sintering temperature required by Barbarossa et al. [21] to obtain approximately 97% dense additive-free (Hf_0.2_Mo_0.2_Ti_0.2_Ta_0.2_Zr_0.2_)B_2_ was significantly higher (1950 °C) than that required in this work (1800 °C) for the preparation of the composite. Therefore, the role played by SiC as a sintering aid was, once more, ascertained.

### 3.3. Oxidation Behavior

The oxidation resistance of the two HEB–SiC ceramics obtained in this work was evaluated based on their behavior during 1 h heat treatment in an air furnace at different temperature conditions, in the range 600–1300 °C. For the sake of comparison, the corresponding additive-free samples were also tested.

As shown in Supplementary Figure S2, HEB_Nb and HEB_Zr specimens markedly changed their appearance upon the oxidation test since their original gray-brown color turned first to gray-blue (700 °C), then to orange (1200–1300 °C). On the other hand, minor differences were observed in SiC-containing samples, which apparently seemed to be less affected by the received heat treatment.

**Figure 11:**
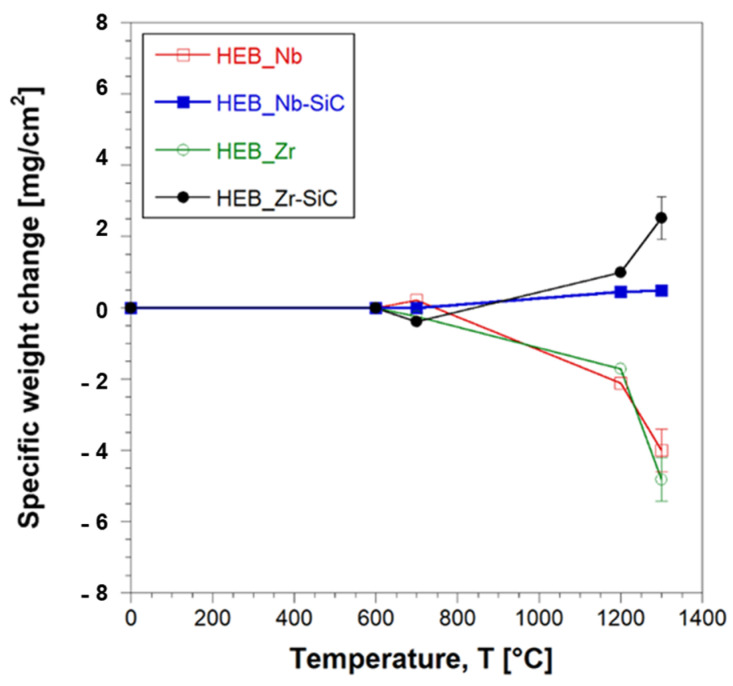
Weight change per surface area of HEB_Nb–SiC HEB_Zr–SiC samples obtained by SPS (T_D_ = 1800 °C, HR = 200 °C/min, t_D_ = 20 min, P = 20 MPa) during oxidation tests carried out at different temperatures in an air furnace. Data relative to additive-free HEB_Nb and HEB_Zr are also plotted, for the sake of comparison.

Figure 11 shows that negligible weight changes occurred in samples heat treated up to 700 °C, regardless of the presence of the additive. Conversely, the latter played a key role when these ceramics were exposed to higher thermal levels. Indeed, pure HEBs lost progressively their weight as the temperature was increased to 1200 °C and, above all, 1300 °C. This feature could be ascribed to the formation of some volatile oxidation products, as discussed later. In contrast, the presence of SiC apparently inhibited such volatilization phenomena since the composite ceramics, particularly HEB_Zr–SiC, displayed a gradual mass increase with temperature.

**Figure 12:**
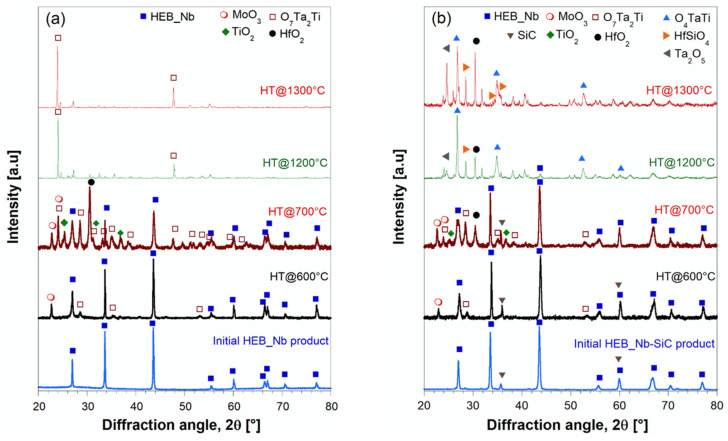
XRD patterns of HEB_Nb (**a**) and HEB_Nb–SiC (**b**) samples after being heat treated in an air furnace at different temperatures. Apart from HEB and SiC, the other crystalline phases identified by XRD analysis in the oxidized samples and the related COD [35] cards are MoO_3_ (1,538,963), O_7_Ta_2_Ti (4,343,510), O_4_TaTi (2,002,580), HfO_2_ (1,528,988), TiO_2_ (9,004,144), Ta_2_O_5_ (2,102,123), HfSiO_4_ (9,000,852), and SiC (1,011,031).

Compositional changes occurring on the surface of the samples annealed at various temperatures were evaluated by XRD analysis. The results obtained with HEB_Nb and HEB_Nb–SiC systems are shown in Figure 12. Up to 600 °C, the composition remained basically unaltered, except for the presence of very small peaks associated to MoO_3_ and TiTa_2_O_7_, which provided an indication of the incipient oxidation of the two ceramics. When temperature was raised to 700 °C, the XRD patterns changed markedly, particularly for the SiC-free material. Under such conditions, the MoO_3_ and TiTa_2_O_7_ peak intensities increased, while two additional oxides (HfO_2_ and TiO_2_) were detected by this analysis, to testify to the oxidation progress. The same oxides were also found on the surface of the HEB_Nb–SiC specimen, while their XRD peaks, particularly those of HfO_2_, were much less intense, while HEB was still the dominant phase. The latter finding provides the first evidence of the beneficial presence of SiC in the ceramic. The lower weight changes observed up to 700 °C (Figure 11) indicated that up to this stage, oxidation phenomena were confined to a small sample volume. As specimens were heat treated at 1200 °C, the two systems behaved quite differently. As for HEB_Nb, the mixed oxide ascribed to TiTa_2_O_7_ was the only phase clearly detected by XRD. An almost identical situation was also encountered at 1300 °C. According to previous works [8], some of the formed oxides, such as MoO_3_, are volatile, so that they tend to leave the sample at such temperature levels. This feature can explain the corresponding weight decrease observed for this system (Figure 11). Interestingly, Gild et al. [8] found that the HEB_Nb and HEB_Zr samples they produced gained weight during oxidation experiments, which is in contrast with results obtained in this work (Figure 11). Such discrepancies can be likely explained by the rather different relative densities of the sintered samples considered in the two studies. Indeed, while a relatively higher porosity was present in samples exposed to the oxidation environment in [8] (relative densities of 92.2 and 92.4% for HEB_Nb and HEB_Zr, respectively), denser samples were characterized in the present work (97.4 ± 0.3 and 96.5 ± 0.7 for HEB_Nb and HEB_Zr, respectively). Therefore, oxygen was allowed to diffuse more easily into the bulk of samples in [8] to generate larger quantities of solid oxides (HfO_2_, ZrO_2_, etc.), which were responsible for the reported weight gain of the oxidized ceramics. In contrast, the higher densification level achieved in the present study made oxygen diffusion across the sample volume more difficult, so that oxidation phenomena were mainly localized on the material surface, so that the prevailing effect was the generation of volatile oxides (MoO_3_, etc.), which caused the observed weight loss. Another difference, which could also play a role in this regard, is that Gild et al. [8] conducted oxidation tests in flowing air, which was not the case in the present study.

SiC introduction produced major effects on the oxidation behavior of the ceramic when operating at 1200–1300 °C. Correspondingly, some silicate phases, either crystalline, such as HfSiO_4_, detected by XRD analysis (Figure 12b), or amorphous, were formed. These latter phases were expected to incorporate various oxides, including the volatiles ones, so that they were not allowed to escape from the sample. This fact could be readily correlated to the mass gained by HEB_Nb–SiC (Figure 11). A similar behavior was also observed with the Zr-containing system. Therefore, the addition of SiC to HEB phases clearly impeded the progressive loss of volatile oxides formed when these ceramics were exposed to oxidation environments at high temperatures.

The results shown in Figure 11 evidence that the HEB_Zr–SiC samples increased their weight more than the HEB_Nb–SiC counterparts when subjected to temperatures equal to or exceeding 1200 °C. Detailed theoretical and experimental studies, rather complicated for such multi-component systems, are needed to provide a clear explanation of such an outcome. Even though the scope of this work was to evidence the effect produced by the introduction of SiC on the oxidation properties of HEB_Zr and HEB_Nb, some general considerations can be also made regarding the different behaviors manifested by the two SiC-containing ceramics. Oxidation resistance is well known to depend on both kinetic and thermodynamic aspects. As for the thermodynamic stability of the oxide phases formed by the two composite systems investigated in this work, the higher affinity of zirconium, with respect to niobium, for oxygen can probably explain why the mass gain of HEB_Zr–SiC at elevated temperatures was relatively higher. Nonetheless, as mentioned above, an in-depth dedicated study, also involving kinetic considerations, is required to reach reliable and consistent conclusions on this matter.

### 3.4. Mechanical Properties

Table 3 reports Vickers hardness and fracture toughness values measured for the different SiC-containing and additive-free HEB_Nb and HEB_Zr samples fabricated in this work under the diverse conditions necessary to produce the desired quinary diboride and SiC phases. The available literature data on different HEB–SiC bulk ceramics are also included in this table, along with the corresponding fabrication routes and processing conditions. It is apparent that the introduction of SiC to the HEB_Nb system provided a significant improvement in the K_IC_ value. As expected, the presence of the carbide phase led to the deflection and branching of the cracks formed by Vickers indentation (Figure S5). The lesser was the distance between the SiC phases distributed in the HEB matrix, and the higher was the fracture toughness of the system.

Table 3 also evidences that fracture toughness decreased as the sintering conditions became progressively more severe, i.e., 7.35 MPa m^1/2^ (1800 °C/20 min), 6.23 MPa m^1/2^ (1900 °C/5 min), and 5.36 MPa m^1/2^ (1900 °C/20 min). This finding can be likely ascribed to the grain growth correspondingly taking place, as testified by the increased crystallite size of the HEB phase (cf. Table S3). As for the measured Vickers hardness, it remained roughly the same, in the range 26–27 GPa. A marked improvement in fracture toughness, albeit at a lower level with respect to the HEB_Nb-based system, was also found when adding SiC to the HEB_Zr matrix.

The effect of the introduction of 20 vol.% SiC on the HEB_Nb system was investigated by Zhang et al. [18]. A slightly superior HV value compared with that obtained in this work (28.1 ± 0.9 instead of 27.0 ± 1.7 GPa, respectively) was obtained in the latter study. In this regard, it should be also considered that a relatively higher load, i.e., 1.96 instead of 1 N, was applied in [18], so that the difference in the measured HV values was expected to be larger due to the indentation size effect. Such discrepancy could be likely motivated by the more severe SPS conditions (T_D_ = 2000 °C) adopted to consolidate their BCR powders but also to the lower SiC content (20 instead of 27.7 vol.%) present in the ceramic. In contrast, the measured K_IC_ value was significantly higher in our study. This fact can be ascribed not only to the lower SiC fraction but also to possible negative effects produced by grain growth taking place when the sample was exposed to high sintering temperatures. No further studies can be found in the literature on HEB_Nb–SiC and HEB_Zr–SiC composite formulations. When the comparison was extended to the other similar systems reported in Table 3, it can be stated that the composite ceramics produced in this work by SHS–SPS generally exhibited superior HV and K_IC_ properties. This outcome can be attributed to various causes, first the different nominal composition of the HEB phase and SiC content and the use of alternative synthesis methods for powder preparation (BR and BCR), as well as to the applied sintering conditions. All these aspects were co-responsible for determining the performances of the produced material. For instance, the fact that the secondary SiC phase was directly synthesized in situ (during the SHS and SPS stages), instead of adding it after powder preparation by BR/BCR [16,17,18,20], is expected to be highly beneficial to establish stronger interfaces with the HEB matrix and, in turn, improve the mechanical properties of the ceramic. So far, the only literature study on HEB–SiC composites adopting a similar approach was focused on the direct synthesis by BR of the (V,Ti,Ta,Nb)B_2_–SiC composite starting from V_2_O_5_, TiO_2_, Ta_2_O_5_, Nb_2_O_5_, B_4_C, carbon black, and Si [19]. Nonetheless, apart from the different route and HEB composition, as compared with those considered in this work, Gong et al. [19] addressed their study only to the powder preparation. In any case, as clearly demonstrated in Licheri et al. [36], the co-synthesis of the different phases involved in a ceramic composite provides beneficial effects with respect to their combination as individual constituents.

## 4. Conclusions

Dense (Hf_0.2_Mo_0.2_Ti_0.2_Ta_0.2_Nb_0.2_)B_2_–SiC and (Hf_0.2_Mo_0.2_Ti_0.2_Ta_0.2_Zr_0.2_)B_2_–SiC were synthesized in this work from elemental transition metals, B_4_C, and Si. While the prescribed HEB and SiC phases could not be obtained by the one-step reactive SPS process carried out at 2000 °C/20 min/20 MPa, this goal was reached with the SHS–SPS route. The initial precursors were only partially converted by SHS, while the completion of the synthesis reaction was attained by SPS under the optimized condition of 1800 °C/20 min/20 MPa. The resulting HEB–SiC samples were characterized by relative densities higher than 97% and did not contain any secondary boride phases. Only small amounts of oxides (in the range 1.1–2.4 wt.%) were detected in the sintered product. The introduction of SiC was highly beneficial to improve sintering behavior, mechanical, and oxidation resistance properties, compared with the corresponding additive-free high-entropy borides. The Nb-containing system processed by SPS at 1800 °C/20 min displayed superior fracture toughness (7.35 MPa m^1/2^) compared with the HEB_Zr–SiC system (4.11 MPa m^1/2^). During oxidation tests, SiC played a key role in hindering the volatilization of the formed metal oxides like MoO_3_, so that the significant weight loss occurring in the additive-free ceramics could be minimized.

In addition to the intrinsic benefits induced by the use of this additive, a positive effect was derived by the in situ synthesis of both ceramic constituents during the SHS and SPS stages. This is expected to highly promote the establishment of strong interfaces between the formed phases and, consequently, enhance the performances of resulting material.

## Figures and Tables

**Table 1:** Characteristics of precursors used for the synthesis of HEB_Nb–SiC and HEB_Zr–SiC by R-SPS and SHS.

Reactant	Vendor (Code)	Particle Size (µm)	Purity (%)
Hf	Alfa Aesar, Karlsruhe, Germany (10201)	<44	99.6
Mo	Alfa Aesar (10031)	<149	≥99
Ta	Alfa Aesar (00337)	<44	99.8
Ti	Aldrich, St. Louis, MI, USA (26.849-6)	<149	99.7
Nb	Alfa Aesar (010275)	<44	99.8
Zr	Thermo Scientific, Waltham, MA, USA (00847)	2–3	-
Si	Aldrich (21561-9)	<44	99%
B_4_C	Alfa Aesar (40504)	1–7	99.4

**Table 2:** Particle size characteristics, as determined by laser scattering analysis, of the SHS powders to be consolidated by SPS.

System ID	d_10_ (µm)	d_50_ (µm)	d_90_ (µm)	d[4,3] (µm)
HEB_Nb–SiC	0.155 ± 0.025	0.825 ± 0.215	6.645 ± 1.055	2.185 ± 0.395
HEB_Zr–SiC	0.13 ± 0.01	0.61 ± 0.04	5.715 ± 0.435	1.88 ± 0.15

**Table 3:** Hardness and fracture toughness of HEB_Nb- and HEB_Zr-based ceramics obtained by SPS in this work. The corresponding values reported in the literature for similar HEB–SiC systems are also included, along with the related fabrication method (SHS: self-propagating high-temperature synthesis; BR: borothermal reduction; BCR: boro-carbothermal reduction; SPS: spark plasma sintering; HP: hot pressing), sintering conditions, and sample densities. n.r.: not reported.

System	Fabrication Method	Sintering Conditions (T_D_, t_d_, P)	ρ (%)	HV (Load, N) (GPa)	K_IC_ (MPa m^1/2^)	Reference
(Hf_0.2_Mo_0.2_Ti_0.2_Ta_0.2_Nb_0.2_)–0 vol.%SiC	SHS–SPS	1950 °C/2 min/20 MPa	97.4 ± 0.3	27.0 ± 1.3 (1)	2.61 ± 0.17	This work
(Hf_0.2_Mo_0.2_Ti_0.2_Ta_0.2_Nb_0.2_)–27.7 vol.%SiC	SHS–SPS	1800 °C/20 min/20 MPa	97.2 ± 1.4	26.0 ± 1.0 (1)	7.35 ± 0.66	This work
	SHS–SPS	1900 °C/5 min/20 MPa	98.3 ± 1.3	27.0 ± 1.7 (1)	6.23 ± 0.50	This work
	SHS–SPS	1900 °C/20 min/20 MPa	98.3 ± 1.2	27.0 ± 1.7 (1)	5.36 ± 0.37	This work
(Hf_0.2_Mo_0.2_Ti_0.2_Ta_0.2_Zr_0.2_)B_2_–0 vol.%SiC	SHS–SPS	1950 °C/20 min/20 MPa	96.5 ± 0.7	25.0 ± 1.6 (1)	2.11 ± 0.15	This work
(Hf_0.2_Mo_0.2_Ti_0.2_Ta_0.2_Zr_0.2_)B_2_–27.4 vol.%SiC	SHS–SPS	1800 °C/20 min/20 MPa	97.7 ± 0.6	27.0 ± 1.5 (1)	4.11 ± 0.32	This work
(Hf_0.2_Mo_0.2_Ti_0.2_Ta_0.2_Nb_0.2_)–20 vol.%SiC	BR–SPS	2000 °C/10 min/30 MPa	99.1 ± 0.1	26.2 ± 1.8 (1.96)	4.41 ± 0.21	[18]
	BCR–SPS		100.0 ± 0.5	28.1 ± 0.9 (1.96)	4.25 ± 0.37	
(Hf_0.2_Mo_0.2_Ti_0.2_Zr_0.2_Nb_0.2_)–20 vol.%SiC	BR–SPS	2000 °C/10 min/30 MPa	100.0 ± 0.4	25.8 ± 1.2 (1.96)	4.53 ± 0.66	[18]
	BCR–SPS		98.6 ± 0.2	29.0 ± 1.3 (1.96)	3.80 ± 0.33	
(Hf_0.2_Nb_0.2_Ti_0.2_Ta_0.2_Zr_0.2_)B_2_–10 vol.%SiC	BCR–SPS	1800 °C/10 min/30 MPa	99.47	~20.1 (49)	~5.00	[20]
(Hf_0.2_Nb_0.2_Ti_0.2_Ta_0.2_Zr_0.2_)B_2_–20 vol.%SiC	BCR–SPS	1800 °C/10 min/30 MPa	99.51	~20.7 (49)	~5.20	[20]
	BCR–HP	1800 °C/60 min/n.r.	>99	24.8 ± 1.2 (1.96)	4.85 ± 0.33	[17]
	BCR–SPS	1600–1900 °C/10 min/30 MPa	>97	~23 (49)	~4.7	[16]
(Hf_0.2_Nb_0.2_Ti_0.2_Ta_0.2_Zr_0.2_)B_2_–30 vol.%SiC	BCR–SPS	1800 °C/10 min/30 MPa	99.73	~21.1 (49)	~5.20	[20]

## Data Availability

Data are contained within the article and Supplementary Materials.

## Supplementary Materials

The following supporting information can be downloaded at https://www.mdpi.com/article/10.3390/ma17030718/s1: Figure S1: (a) SEM micrograph along with the corresponding EDS elemental maps and (b) X-EDS pattern of HEB_Nb–SiC powders synthesized by SHS; Figure S2: XRD patterns of HEB_Nb–SiC products obtained by SHS–SPS at different T_D_ values: (a) t_D_ = 5 min and (b) t_D_ = 20 min. The XRD pattern of SHS powders is also included, for comparison; Figure S3: Optical images (complete and detailed view) showing the surface changes of (Hf_0.2_Mo_0.2_Ti_0.2_Ta_0.2_Nb_0.2_)B_2_ and (Hf_0.2_Mo_0.2_Ti_0.2_Ta_0.2_Nb_0.2_)B_2_–SiC samples after oxidation experiments in an air furnace at different temperatures; Figure S4: Optical images (complete and detailed view) showing the surface changes of (Hf_0.2_Mo_0.2_Ti_0.2_Ta_0.2_Zr_0.2_)B_2_ and (Hf_0.2_Mo_0.2_Ti_0.2_Ta_0.2_Zr_0.2_)B_2_–SiC samples after oxidation experiments in an air furnace at different temperatures; Figure S5: Example of SEM images showing the cracks propagated from the indent tips to evaluate the fracture toughness: (a) the HEB_Nb–SiC and (b) the HEB_Zr–SiC systems; Table S1: Phases and quantitative phase analysis results of the HEB_Nb–SiC product obtained by reactive SPS. (SS): solid solution; Table S2: Phases and quantitative phase analysis results of the HEB_Nb–SiC product obtained by SHS; Table S3: Phases and quantitative phase analysis results of the HEB_Nb–SiC products obtained by SPS, at different operating conditions, from SHS powders; Table S4: Phases and quantitative phase analysis results of the HEB_Zr–SiC products obtained by SHS and SPS. References [37,38] are cited in the supplementary materials.
