# Correction: Pakhomova et al. High-Entropy Diborides—Silicon Carbide Composites by Reactive and Non-Reactive Spark Plasma Sintering: A Comparative Study. *Materials* **2024**, *17*, 718

**DOI:** 10.3390/ma17133274

**Published:** 2024-07-03

**Authors:** Ekaterina Pakhomova, Giacomo Cao, Roberto Orrù, Sebastiano Garroni, Paolo Ferro, Roberta Licheri

**Affiliations:** 1 Unità di Ricerca del Consorzio Interuniversitario Nazionale per la Scienza e Tecnologia dei Materiali (INSTM), Dipartimento di Ingegneria Meccanica, Chimica, e dei Materiali, Università degli Studi di Cagliari, via Marengo 2, 09123 Cagliari, Italy; ekaterina.pakhomova@unica.it (E.P.); giacomo.cao@unica.it (G.C.); roberta.licheri@unica.it (R.L.); 2 Dipartimento di Scienze Chimiche, Fisiche, Matematiche e Naturali, Università degli Studi di Sassari, 07100 Sassari, Italy; sgarroni@uniss.it; 3 Dipartimento di Tecnica e Gestione dei Sistemi Industriali, Università di Padova, Stradella S. Nicola 3, 36100 Vicenza, Italy; paolo.ferro@unipd.it

In the original publication [1], there was a mistake in Figure 10. The subfigure (g) Nb is wrong; the correct subfigure should be (g) Zr. The corrected Figure 10 appears below. The authors state that the scientific conclusions are unaffected. This correction was approved by the Academic Editor. The original publication has also been updated.

## Figures and Tables

**Figure 10:**
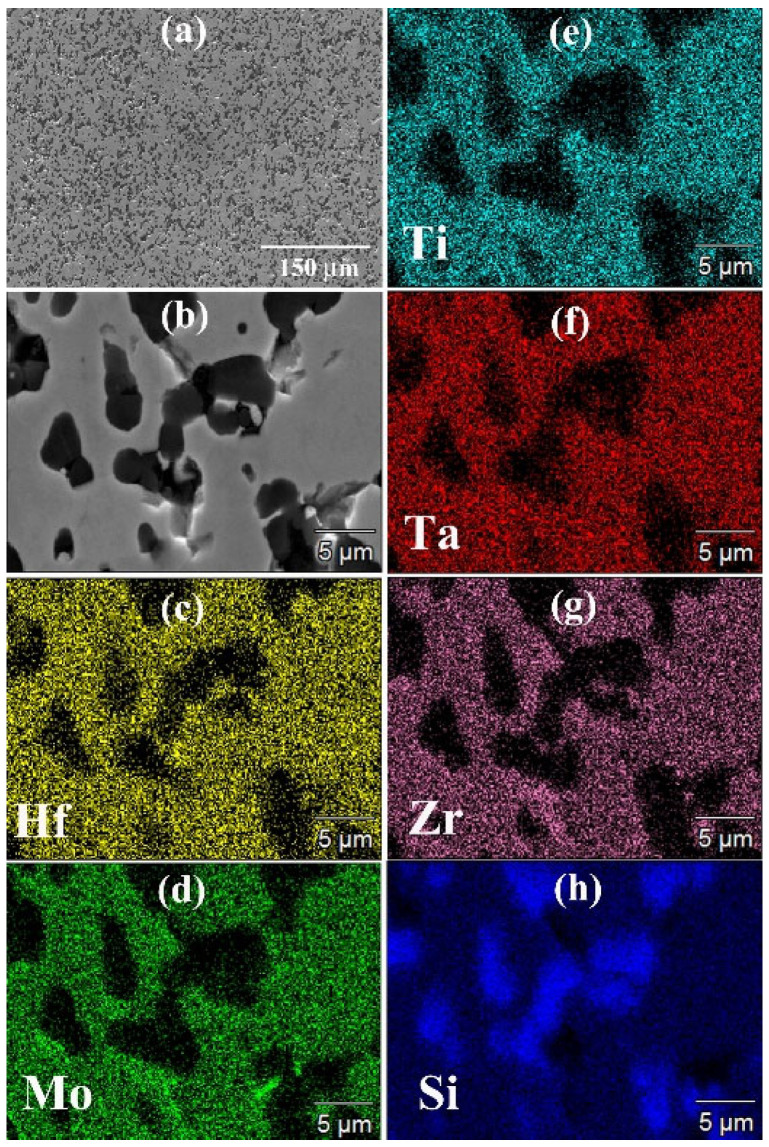
General (**a**) and detailed (**b**) SEM views along with the corresponding Hf (**c**), Mo (**d**), Ti (**e**), Ta (**f**), Zr (**g**), and Si (**h**) EDS maps of the HEB_Zr–SiC sample obtained by SPS (T_D_ = 1800 °C, t_D_ = 20 min, P = 20 MPa, HR = 200 °C/min) from powders synthesized by SHS according to Reaction (1) with Me = Zr.
