# Supplementary material for: High-Entropy Diborides—Silicon Carbide Composites by Reactive and Non-Reactive Spark Plasma Sintering: A Comparative Study
Source: Materials (Basel). 2024 Feb 2;17(3):718. doi: 10.3390/ma17030718 (PMC10856243; doi:10.3390/ma17030718)
Supplement: Supplementary file 1 [file materials-17-00718-s001.zip › materials-2810182-supplementary.pdf]

Supplementary Material for

High-entropy diborides - Silicon carbide composites by reactive and non-reactive spark plasma sintering: a comparative study

Ekaterina Pakhomova, Giacomo Cao, Roberto Orrù,

Sebastiano Garroni, Paolo Ferro, Roberta Licheri

Supplementary **Figures S1-S5** and **Tables S1- S4**

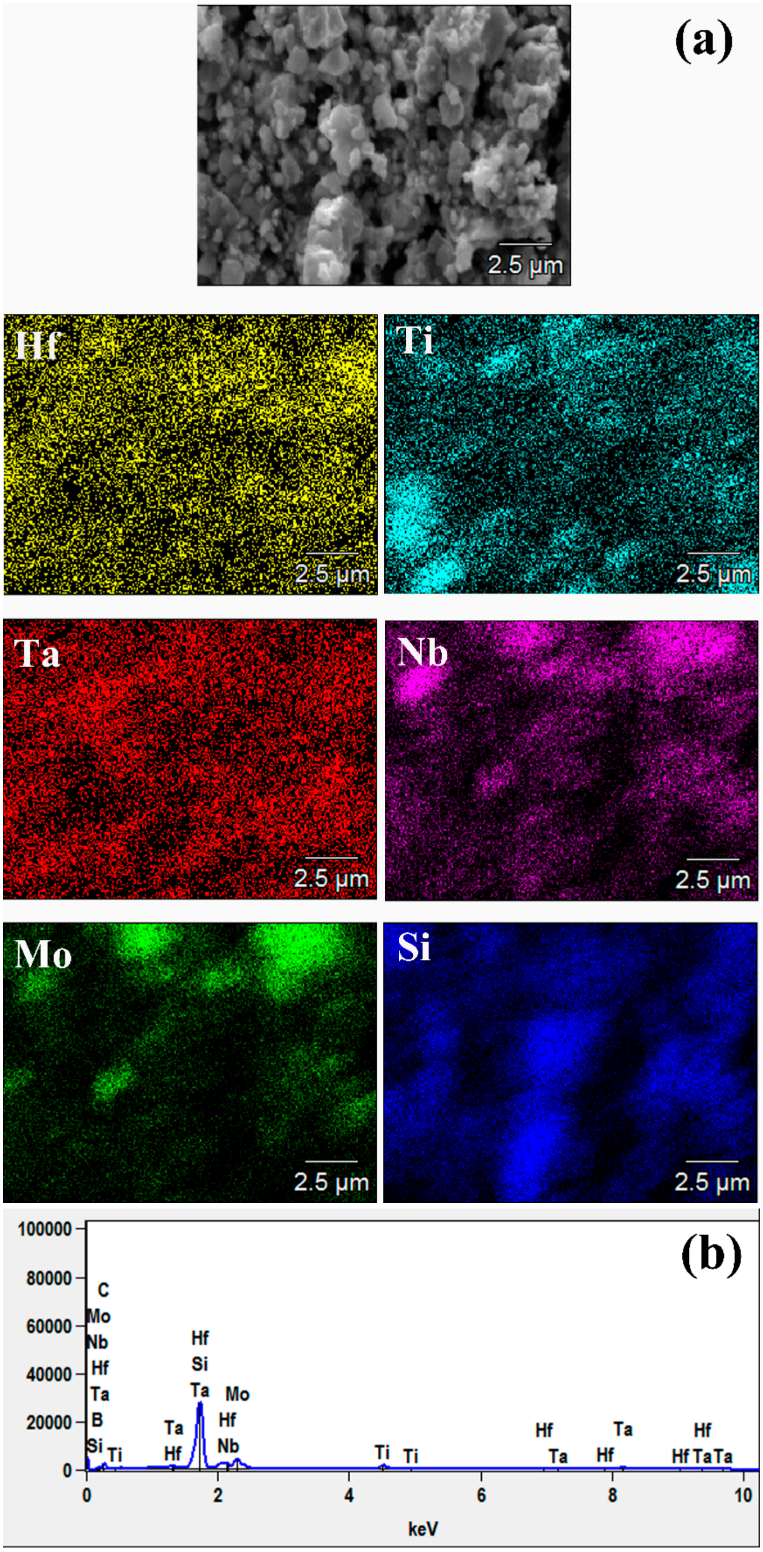

Figure S1. (a) SEM micrograph along with the corresponding EDS elemental maps, and (b) X-EDS pattern of HEB_Nb-SiC powders synthesized by SHS

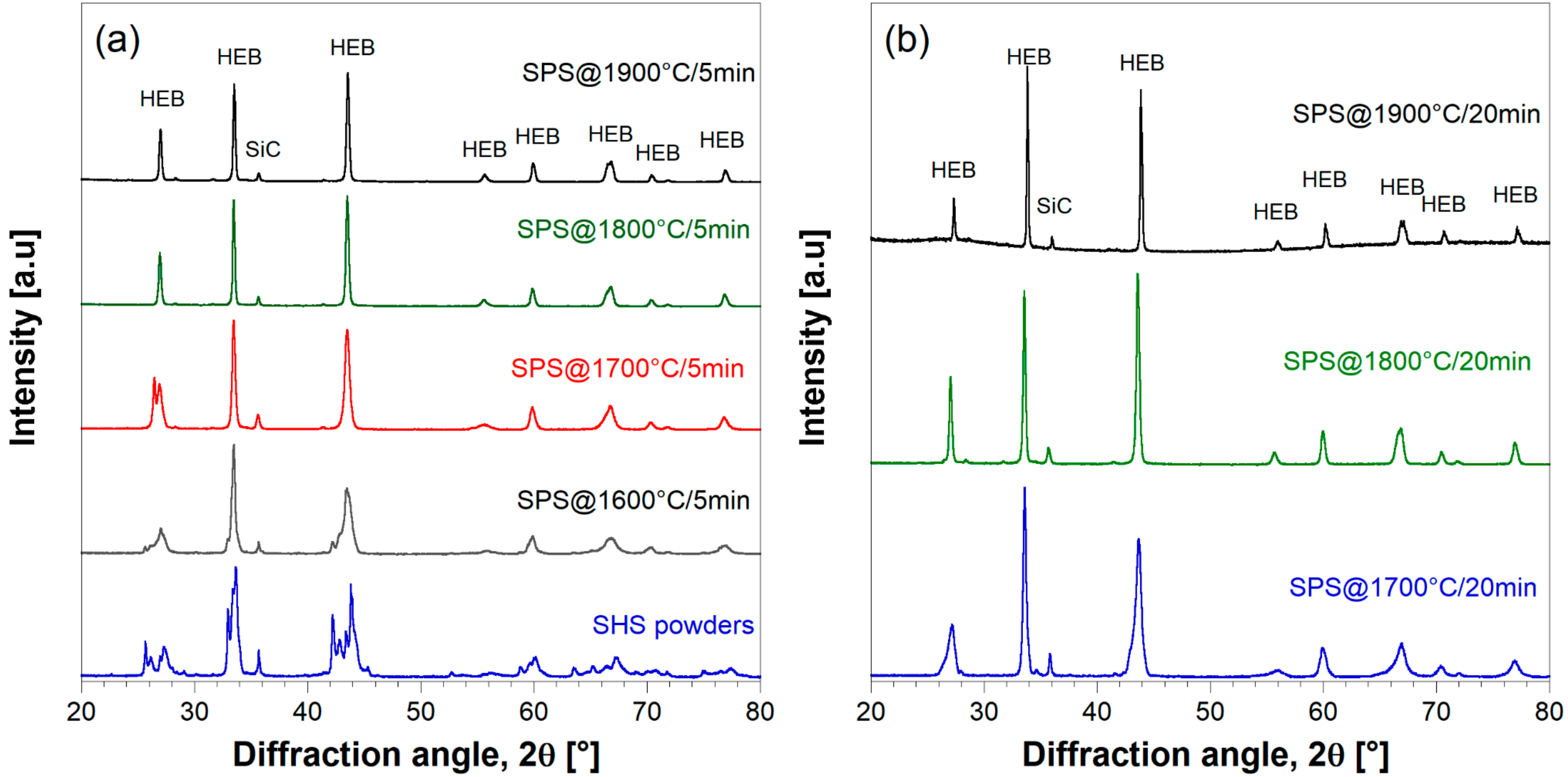

Figure S2. XRD patterns of HEB_Nb-SiC products obtained by SHS-SPS at different T_D values: (a) t_D=5 min, (b) t_D=20 min. The XRD pattern of SHS powders is also included, for comparison.

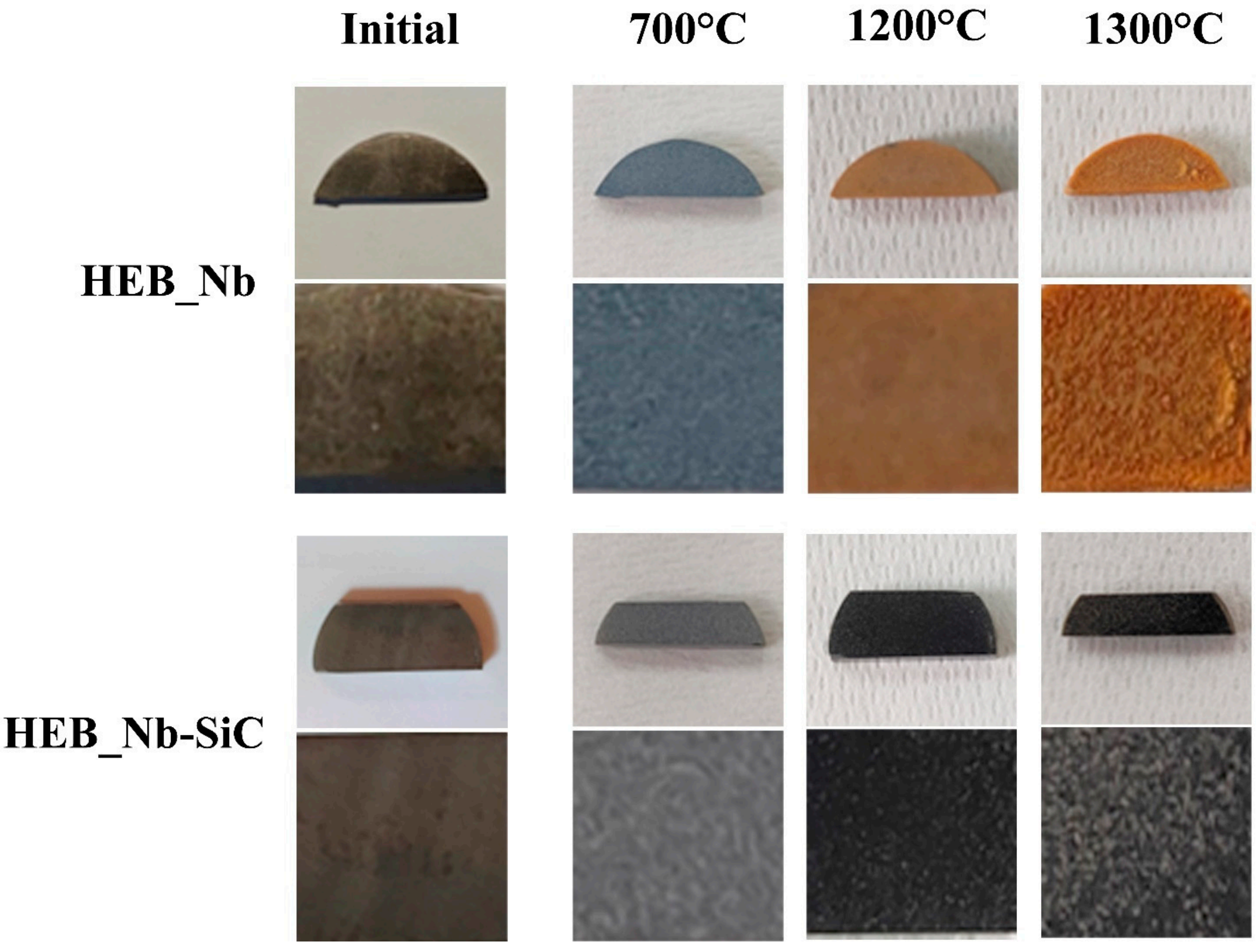

Figure S3. Optical images (complete and detailed view) showing the surface changes of $(Hf_{0.2}Mo_{0.2}Ti_{0.2}Ta_{0.2}Nb_{0.2})B_2$ and $(Hf_{0.2}Mo_{0.2}Ti_{0.2}Ta_{0.2}Nb_{0.2})B_2$-SiC samples after oxidation experiments in air furnace at different temperatures.

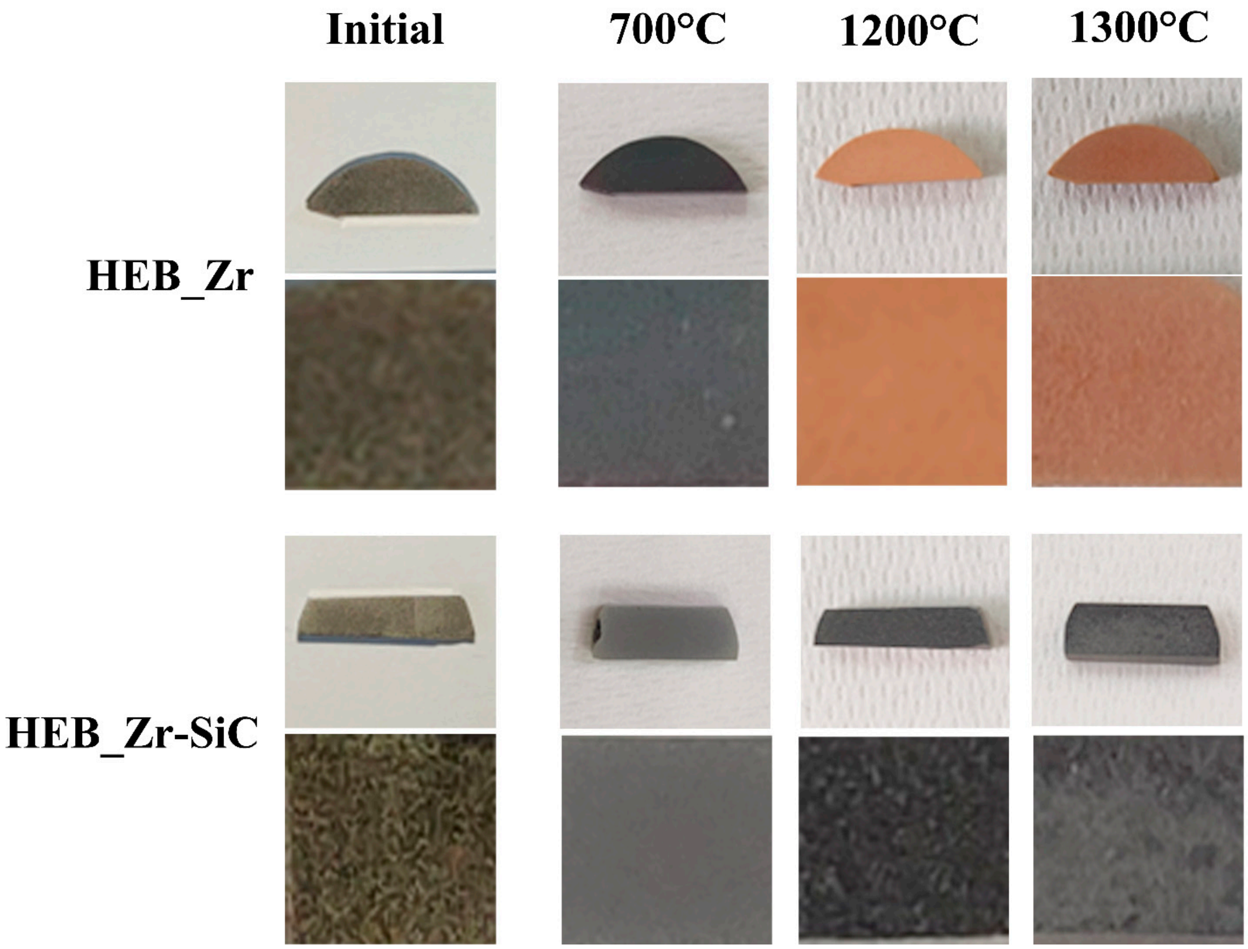

Figure S4. Optical images (complete and detailed view) showing the surface changes of $(Hf_{0.2}Mo_{0.2}Ti_{0.2}Ta_{0.2}Zr_{0.2})B_2$ and $(Hf_{0.2}Mo_{0.2}Ti_{0.2}Ta_{0.2}Zr_{0.2})B_2$-SiC samples after oxidation experiments in air furnace at different temperatures.

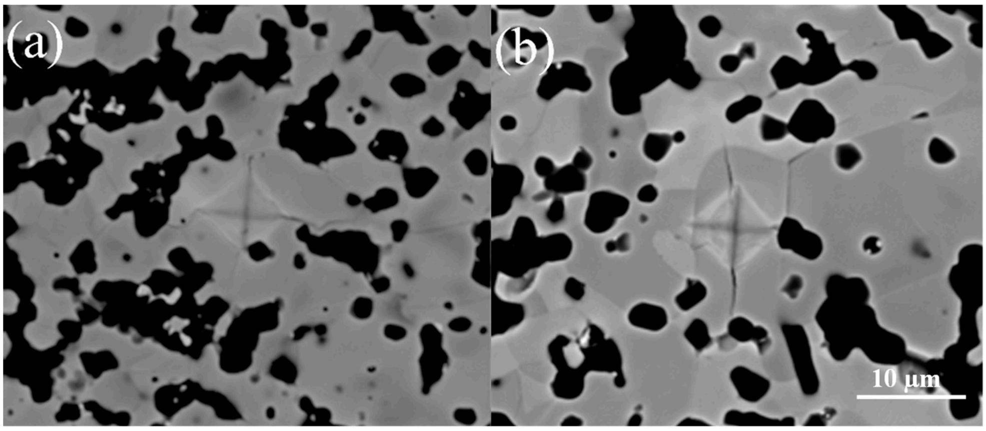

Figure S5. Example of SEM images showing the cracks propagated from the indent tips used to evaluate the fracture toughness of (a) the HEB_Nb-SiC, and (b) HEB_Zr-SiC samples.

Table S1-S4

Cif files from the Crystallography Open Database (COD) available at https://www.crystallography.net/cod/index.php, have used for the Rietveld refinement **[37].**

Bragg reflections belonging to the HEB_Nb and HEB_Zr solid solution phases have been interpolated modifying the cif file used for the system reported in the manuscript of Tallarita el al. **[38].**

For each phase the COD references are here below reported:

SiC: #1011031, MoB_2: #1510765, $(Ta_{0.5}Ti_{0.5})B_2$: #1510835, NbB_2: #1510778, TaB_2: #1510843, TiB_2: # 2002799, HfB_2: # 1510711, $(MoTi)B_4$: # 1510760, $MoSi_2$: # 1010282, C: # 1200017, SiO_2: # 1532513, Si: # 1526655, B_4C: # 4124697, HfO_2: # 1528988, ZrB_2: # 1510856.

Table S1. Phases and quantitative phase analysis results of the **HEB_Nb-SiC** product obtained by Reactive SPS. (SS): Solid Solution

R-SPS (2000°C, 20 min)									
**Phase**	**wt. (%)**	**a (Å)**	**b (Å)**	**c (Å)**	**β (°)**	**Symmetry**	**Space Group**	**Crystallite size (Å)**	**Microstrain**
$(Hf_{0.2}Mo_{0.2}Ti_{0.2}Ta_{0.2}Nb_{0.2})B_2$ (SS)-1	20.4	3.0974	-	3.3196	-	Hexagonal	P6/mmm	1104	4.59 E-4
$(Hf_{0.2}Mo_{0.2}Ti_{0.2}Ta_{0.2}Nb_{0.2})B_2$ (SS)-2	20.3	3.0904	-	3.3032	-	Hexagonal	P6/mmm	1092	1.14 E-3
SiC	12.8	4.3529	-	3.3196	-	Cubic	F-43m	>1500	1.24 E-3
MoB_2	16.7	3.1030	-	3.2118	-	Hexagonal	P6/mmm	988	1.53 E-4
NbB_2	15.1	3.0746	-	3.2620	-	Hexagonal	P6/mmm	1006	1.82 E-3
$(Ta_{0.5}Ti_{0.5})B_2$	14.7	3.0496	-	3.2381	-	Hexagonal	P6/mmm	991	1.58 E-4

Table S2. Phases and quantitative phase analysis results of the **HEB_Nb-SiC** product obtained by SHS

SHS									
**Phase**	**wt. (%)**	**a (Å)**	**b (Å)**	**c (Å)**	**β (°)**	**Symmetry**	**Space Group**	**Crystallite size (Å)**	**Microstrain**
$(Hf_{0.2}Mo_{0.2}Ti_{0.2}Ta_{0.2}Nb_{0.2})B_2$	14.9	3.1051	-	3.4182	-	Hexagonal	P6/mmm	845	4.93 E-3
SiC	11.5	4.3614	-	-	-	Cubic	F-43m	>1500	3.09 E-3
TaB_2	14.6	3.0769	-	3.2720	-	Hexagonal	P6/mmm	1125	2.15 E-3
TiB_2	13.4	3.0566	-	3.2437	-	Hexagonal	P6/mmm	1382	4.21E-3
NbB_2	12.8	3.1014	-	3.3110	-	Hexagonal	P6/mmm	1265	1.58 E-3
MoB_2	9.5	2.9870	-	3.1920	-	Hexagonal	P6/mmm	1038	5.56 E-3
HfB_2	9.0	3.1412	-	3.4739	-	Hexagonal	P6/mmm	1227	1.21 E-3
$(MoTi)B_4$	3.9	3.2788	-	2.6036	-	Hexagonal	P6/mmm	799	1.46 E-3
$MoSi_2$	2.9	3.2088	-	7.8529	-	Tetragonal	I4/mmm	1077	9.54 E-4
C	2.9	2.4646	-	6.7332	-	Hexagonal	P63mc	1030	1.28 E-3
SiO_2	1.7	7.0669	12.3077	7.1960	120.4	Monoclinic	C2/c:b1	896	1.34 E-3
Si	1.6	5.4375	-	-	-	Cubic	Fd-3m:1	972	1.43 E-3
B_4C	1.3	5.6010	-	12.2410	-	Trigonal	R-3m:H	978	1.1 E-4

Table S3. Phases and quantitative phase analysis results of the **HEB_Nb-SiC** products obtained by SPS, at different operating conditions, from SHS powders.

SPS (1800°C, 20 min)									
**Phase**	**wt. (%)**	**a (Å)**	**b (Å)**	**c (Å)**	**β (°)**	**Symmetry**	**Space Group**	**Crystallite size (Å)**	**Microstrain**
$(Hf_{0.2}Mo_{0.2}Ti_{0.2}Ta_{0.2}Nb_{0.2})B_2$	83.1	3.0887	-	3.3035	-	Hexagonal	P6/mmm	1089	1.33 E-3
SiC	13.6	4.3605	-	-	-	Cubic	F-43m	>1500	1.43 E-3
HfO_2	2.4	5.1201	5.1748	5.2851	99.1	Monoclinic	P21/c:b1	974	3.33 E-3
C	0.9	2.4584	-	6.7480	-	Hexagonal	P63mc	1005	2.72E-4

SPS (1900°C, 5 min)									
**Phase**	**wt. (%)**	**a (Å)**	**b (Å)**	**c (Å)**	**β (°)**	**Symmetry**	**Space Group**	**Crystallite size (Å)**	**Microstrain**
$(Hf_{0.2}Mo_{0.2}Ti_{0.2}Ta_{0.2}Nb_{0.2})B_2$	85.6	3.0887	-	3.3035	-	Hexagonal	P6/mmm	1319	1.52 E-4
SiC	13.0	4.3605	-	-	-	Cubic	F-43m	>1500	1.03 E-3
HfO_2	1.4	5.1201	5.1748	5.2851	99.1	Monoclinic	P21/c:b1	993	6.05 E-4

SPS (1900°C, 20 min)									
**Phase**	**wt. (%)**	**a (Å)**	**b (Å)**	**c (Å)**	**β (°)**	**Symmetry**	**Space Group**	**Crystallite size (Å)**	**Microstrain**
$(Hf_{0.2}Mo_{0.2}Ti_{0.2}Ta_{0.2}Nb_{0.2})B_2$	85.2	3.0891	-	3.3027	-	Hexagonal	P6/mmm	1517	1.04 E-4
SiC	13.7	4.3611	-	-	-	Cubic	F-43m	>1500	1.31 E-3
HfO_2	1.1	5.1248	5.1777	5.2948	99.3	Monoclinic	P21/c:b1	1069	1.21 E-4

Table S4. Phases and quantitative phase analysis results of the **HEB_Zr-SiC** products obtained by SHS, and SPS.

SHS									
**Phase**	**wt. (%)**	**a (Å)**	**b (Å)**	**c (Å)**	**β (°)**	**Symmetry**	**Space Group**	**Crystallite size (Å)**	**Microstrain**
$(Hf_{0.2}Mo_{0.2}Ti_{0.2}Ta_{0.2}Zr_{0.2})B_2$	11.9	3.1091	-	3.4226	-	Hexagonal	P6/mmm	1032	5.28 E-3
$(MoTi)B_4$	3.4	3.2902	-	2.5892	-	Hexagonal	P6/mmm	845	3.66 E-3
ZrB_2	13.7	3.0969	-	3.3188	-	Hexagonal	P6/mmm	1430	3.52 E-3
TaB_2	15.2	3.0758	-	3.2806	-	Hexagonal	P6/mmm	1197	2.67 E-3
HfB_2	10.7	3.1405	-	3.4731	-	Hexagonal	P6/mmm	1275	2.45 E-3
SiC	10.9	4.3613	-	-	-	Cubic	F-43m	>1500	5.55 E-4
MoB_2	10.8	2.9741	-	3.1920	-	-	P6/mmm	1039	2.69 E-3
TiB_2	14.6	3.0567	-	3.2424	-	-	P6/mmm	1383	4.17E-3
$MoSi_2$	2.6	3.2086	-	7.8408	-	-	I4/mmm	1062	1.24 E-3
SiO_2	1.9	7.0685	12.3074	7.1961	120.4	Monoclinic	C2/c:b1	799	7.45 E-3
C	1.8	2.4677	-	6.7188	-	Hexagonal	P63mc	1030	1.17 E-3
Si	1.3	5.4378	-	-	-	Cubic	Fd-3m:1	972	2.32 E-3
B_4C	1.2	5.6005	-	12.2459	-	Trigonal	R-3m:H	999	7.86 E-4

SPS (1800°C, 20 min)									
**Phase**	**wt. (%)**	**a (Å)**	**b (Å)**	**c (Å)**	**β (°)**	**Symmetry**	**Space Group**	**Crystallite size (Å)**	**Microstrain**
$(Hf_{0.2}Mo_{0.2}Ti_{0.2}Ta_{0.2}Zr_{0.2})B_2$	84.4	3.0976	-	3.3628	-	Hexagonal	P6/mmm	1229	8.30 E-4
SiC	14.1	4.3593	-	-	-	Cubic	F-43m	>1500	4.50 E-4
HfO_2	1.5	5.1958	5.2193	5.3845	99.2	Monoclinic	P21/c:b1	1069	1.21 E-4
